# A Peptide Vaccine Candidate Tailored to Individuals' Genetics Mimics the Multi-Targeted T Cell Immunity of COVID-19 Convalescent Subjects

**DOI:** 10.3389/fgene.2021.684152

**Published:** 2021-06-23

**Authors:** Eszter Somogyi, Zsolt Csiszovszki, Levente Molnár, Orsolya Lőrincz, József Tóth, Sofie Pattijn, Jana Schockaert, Aurélie Mazy, István Miklós, Katalin Pántya, Péter Páles, Enikő R. Tőke

**Affiliations:** ^1^Treos Bio Ltd., London, United Kingdom; ^2^Treos Bio Zrt, Veszprém, Hungary; ^3^ImmunXperts Société Anonyme, A Nexelis Group Company, Gosselies, Belgium; ^4^Alfréd Rényi Institute of Mathematics, Eötvös Loránd Research Network, Budapest, Hungary

**Keywords:** global vaccine, HLA-genotype, ethnic diversity, SARS-CoV-2 immunity, *in silico* clinical trial

## Abstract

Long-term immunity to coronaviruses likely stems from T cell activity. We present here a novel approach for the selection of immunoprevalent SARS-CoV-2-derived T cell epitopes using an *in silico* cohort of HLA-genotyped individuals with different ethnicities. Nine 30-mer peptides derived from the four major structural proteins of SARS-CoV-2 were selected and included in a peptide vaccine candidate to recapitulate the broad virus-specific T cell responses observed in natural infection. PolyPEPI-SCoV-2-specific, polyfunctional CD8^+^ and CD4^+^ T cells were detected in each of the 17 asymptomatic/mild COVID-19 convalescents' blood against on average seven different vaccine peptides. Furthermore, convalescents' complete HLA-genotype predicted their T cell responses to SARS-CoV-2-derived peptides with 84% accuracy. Computational extrapolation of this relationship to a cohort of 16,000 HLA-genotyped individuals with 16 different ethnicities suggest that PolyPEPI-SCoV-2 vaccination will likely elicit multi-antigenic T cell responses in 98% of individuals, independent of ethnicity. PolyPEPI-SCoV-2 administered with Montanide ISA 51 VG generated robust, Th1-biased CD8^+^, and CD4^+^ T cell responses against all represented proteins, as well as binding antibodies upon subcutaneous injection into BALB/c and hCD34^+^ transgenic mice modeling human immune system. These results have implications for the development of global, highly immunogenic, T cell-focused vaccines against various pathogens and diseases.

## Introduction

The pandemic caused by the novel coronavirus SARS-CoV-2 is still evolving after its outbreak in late 2019, reaching second/third peak in a single year. After demonstration of high protective efficacy against symptomatic COVID-19 in large phase III studies, the first vaccines are rapidly being approved for emergency use (Forni et al., 2021). Both the approved vaccines and the numerous vaccine candidates under clinical development are predominantly designed to generate neutralizing antibodies against the viral Spike (S) protein (WHO, 2020). But lessons learned from the SARS and MERS epidemic as well as COVID-19 pandemic indicate potential challenges (Altmann and Boyton, 2020; Green, 2020; Hellerstein, 2020; Peiris and Leung, 2020). Due to waning antibody responses and continuously arising mutations in the S protein, long-term durability of protection remains unknown (Callaway, 2021; Williams and Burgers, 2021). However, T cell responses against coronavirus proteins can last for over a decade, as demonstrated for MERS and SARS, and data collected till today for SARS-COV-2 seem to support this expectation (Channappanavar et al., 2014b; Le Bert et al., 2020; Schwarzkopf et al., 2021).

Importantly, virtually all subjects with a history of SARS-CoV-2 infection mount T cell responses against the virus, including seronegatives and subjects with severe disease (Grifoni et al., 2020; Hellerstein, 2020; Peng et al., 2020; Zou et al., 2020). Moreover, correlation between defective T cell responses and COVID-19 severity was observed (Diao et al., 2020). Higher CD8^+^ T cell counts were also associated with improved overall survival in cancer patients hospitalized for COVID-19 (Huang et al., 2021).

T cell responses are diverse, recognizing 30–40 SARS-CoV-2-epitopes in each person (Tarke et al., 2021). They are directed against the whole antigenic repertoire of the virus, and less dominated by the S-protein (Nelde et al., 2020; Sekine et al., 2020; Tarke et al., 2021). This diversity is associated with asymptomatic/mild disease and likely confers protection against viral escape by mutations.

The first COVID-19 vaccines, while engender robust humoral responses, have mixed potential for inducing CD8^+^ T cell responses (Anderson et al., 2020; Ewer et al., 2020; Jackson et al., 2020; Sahin et al., 2020; Zhang et al., 2021). Multi-epitope CD8^+^ T cell responses against the S protein, as revealed for two studies, were obtained for only a fraction of subjects (24–60%) (Ewer et al., 2020; Sahin et al., 2020). Multi-epitope responses against multiple viral antigens could be theoretically elicited by vaccines using whole virus material, but the assessment of cellular immune reactions was not included in their studies (Zhang et al., 2021).

Therefore, strategies to better mimic the heterogeneity of multi-specific T cell immunity caused by the natural infection would be required to leverage the vital role of both CD8^+^ and CD4^+^T cell responses in reducing the impact of COVID-19 and potentially provoking long-term immune responses (Dan et al., 2021).

The core problem that afflicts T cell-epitope selection, however, is that each human has a unique immune response profile to pathogens. Indeed, for SARS-CoV-2, the infection or the disease course varies according to the genetic diversity represented by different ethnicities and human leukocyte antigen (HLA) alleles, however, the reason is not yet well-understood (Aldridge et al., 2020; Nguyen et al., 2020; Pan et al., 2020; Poland, 2020; Mohammadpour et al., 2021). HLA alleles are the molecular determinants of antigen-specific T cell activation, to kill infected cells. Each human has six major HLA class I and eight major HLA class II alleles, therefore larger populations have hundreds of different alleles and their numerous combinations in each HLA-genotype. As a result, each person's T cells recognize 30–40 epitopes derived from SARS-CoV-2 and only a fraction of them are shared between convalescents, as recently reported by Tarke et al. in a very comprehensive study (Tarke et al., 2021). To capture this heterogeneity during a global SARS-CoV-2 T cell focused vaccine design effort, epitope mapping based on limited number of frequent HLA alleles has been used widely (Ferretti et al., 2020; Nelde et al., 2020). However, in reality, these epitope mapping studies have a low yield (cca. 10%) in terms of confirmed T cell response in HLA-matched subjects (Nelde et al., 2020; Tarke et al., 2021). Therefore, actionable strategies to target not alleles but individuals and ethnic populations are required. We hypothesize that all HLA alleles (HLA genotype) of a subject regulate immune responses capable of killing infected cells, therefore we propose epitope mapping that involves real-subjects with complete HLA-genotype instead of single HLA alleles split from the complexity of allele combinations.

We present here a novel, computer-aided approach for the selection of immunogenic peptides using an ethnically diverse *in silico* human cohort of individuals with complete HLA genotypes. We selected multiple, so called Personal Epitopes (PEPIs, restricted to multiple HLA alleles of a person) shared among high proportion of subjects in each ethnic group of this model population. PolyPEPI-SCoV-2 contains 9 peptides and targets all four major structural proteins of SARS-CoV-2. We demonstrated, that T cells against each selected epitopes were present in majority of COVID-19 convalescent subjects tested, and the frequency was in good agreement with the frequency determined for the *in silico* cohort. More importantly we found, that subjects' complete HLA-genotype influenced their peptide-specific anti-SARS-CoV-2 immune responses, as hypothesized. Immunogenicity and safety of the designed candidate vaccine were confirmed in two mouse models, resulting in the induction of robust CD8^+^ and CD4^+^ T cell responses, against all four targeted SARS-CoV-2 proteins. Our novel approach enables, for the first-time, computational determination of the epitopes that immune systems of individuals in large cohorts can respond to, likely an indispensable tool for both the design of a global vaccine and for post-vaccination surveillance.

## Materials and Methods

### Donors

Donors were recruited based on their clinical history of SARS-CoV-2 infection. Blood samples were collected from convalescent individuals (*n* = 15) at an independent medical research center in The Netherlands under an approved protocol (NL57912.075.16.) or collected by PepTC Vaccines Ltd (*n* = 2). Sera and PBMC samples from non-exposed individuals (*n* = 10) were collected before 2018 and were provided by Nexelis-IMXP (Belgium). All donors provided written informed consent. The study was conducted in accordance with the Declaration of Helsinki. Blood samples from COVID-19 convalescent patients (*n* = 17; 16 with asymptomatic/mild disease and one with severe disease) were obtained 17–148 days after symptom onset. Surprisingly, one positive IgM antibody response was found among the healthy donors, which was excluded from further analysis. Demographic and baseline information of the subjects are provided in Supplementary Table 1. HLA genotyping of the convalescent donor patients from The Netherlands was done by IMGM laboratories GmbH (Martinsried, Germany) using next-generation sequencing. This cohort uses a total of 46 different HLA class I alleles (15 HLA-A^*^, 18 HLA-B^*^, and 13 HLA-C^*^) and 35 different HLA class II alleles (14 DRB1, 12 DQB1, and 9 DPB1). HLA-genotype data of the subjects is provided in Supplementary Table 2.

#### *In silico* Human Cohorts

##### Model Population (n = 433)

The Model Population is a cohort of 433 individuals, representing several ethnic groups worldwide, for whom complete HLA class I genotypes were available (2 × HLA-A, 2 × HLA-B, 2 × HLA-C). The Model Population was assembled from 90 Yoruban African (YRI), 90 European (CEU), 45 Chinese (CHB), 45 Japanese (JPT), 67 subjects with mixed ethnicity (US, Canada, Australia, New Zealand), and 96 subjects from an HIV database (MIX). HLA genotypes were determined using PCR techniques, Affymetrix 6.0 and Illumina 1.0 Million SNP mass arrays, and high-resolution HLA typing of the six HLA genes by Reference Strand-mediated Conformational Analysis (RSCA) or sequencing-based typing (SBT). This cohort uses a total of 152 different HLA class I alleles (49 HLA-A^*^, 71 HLA-B^*^ and 32 HLA-C^*^) representative for 97.4% of the alleles documented in the current global Common, Intermediate and Well-Documented (CIWD) database, well-representing also major ethnicities (database 3.0 released 2020) (Supplementary Table 3) (Hurley et al., 2020). The frequency of the A^*^, B^*^, and C^*^ alleles of the Model population correlates with the frequency documented for >8 million HLA-genotyped subjects of the CIWD database (*R* = 0.943, 0.869, 0.942, respectively, *p* < 0.00001) (Supplementary Figure 1).

##### HLA Class II Cohort (n = 356)

A second cohort of 356 individuals with characterized HLA class II genotypes (2 × HLA-DRB, 2 × HLA-DP, and 2 × HLA-DQ) at four-digit allele resolution was obtained from the dbMHC database, an online available repository operated by the National Center for Biotechnology Information (NCBI) (Helmberg et al., 2004). HLA genotyping was performed by SBT. This cohort uses a total of 150 different HLA class II alleles (41 DRB1, 66 DQB1, and 43 DPB1).

##### Large, US Cohort (n = 16,000)

The database comprising anonymized HLA genotype data from 16,000 individuals was created by obtaining 1,000 donors from each of 16 ethnic groups (500 male and 500 female) from the National Marrow Donor Program (NMDP) (Gragert et al., 2013). The 16 ethnic groups were: African, African American, Asian Pacific Islander, Filipino, Black Caribbean, Caucasian, Chinese, Hispanic, Japanese, Korean, Native American Indian, South Asian, Vietnamese, US, Mideast/North coast of Africa, Hawaiian, and other Pacific Islander. The ethnic groups represented in this large US cohort covers the composition of the global population but they were not weighted for their global representativeness (we intentionally used *n* = 1,000 subjects for each ethnicity).^1^ HLA genotyping was performed by NMDP recruitment labs using sequence-specific oligonucleotide (SSO) and sequence specific primer (SSP) methods with an average “typing resolution score” >0.7. This cohort uses a total of 497 different HLA class I alleles (136 HLA-A^*^ 240 HLA-B^*^ and 121 HLA-C^*^) representative for 99.8% of the alleles documented in the current global Common, Intermediate and Well-Documented (CIWD) database (database 3.0 released in 2020) (Hurley et al., 2020) and 140 HLA class II alleles (105 DRB1 and 35 DQB1, DPB1 was not available). HLA-alleles covered by this cohort are provided in Supplementary Table 4.

### Animals

#### CD34^+^ Transgenic Humanized Mouse (Hu-mouse)

*Female* NOD/Shi-scid/IL-2Rγ null immunodeficient mice (Charles River Laboratories, France) were humanized using hematopoietic stem cells (CD34^+^) isolated from human cord blood. Only mice with a humanization rate (hCD45/total CD45) >50% were used during the study. Experiments were carried out with 20–23-week-old female mice.

#### BALB/c Mouse

Experiments were carried out with 6–8 week old female BALB/c mice (Janvier, France).

### Vaccine Design

#### Tailoring PolyPEPISCoV-2 to SARS-CoV-2 Genetics

SARS-CoV-2 structural proteins (S, N, M, E) were screened and nine different 30-mer peptides were selected during a multi-step process. First, sequence diversity analysis was performed (as of 28 March 2020 in the NCBI database).^2^ The accession IDs were as follows: **NC_045512.2**, MN938384.1, MN975262.1, MN985325.1, MN988713.1, MN994467.1, MN994468.1, MN997409.1, MN988668.1, MN988669.1, MN996527.1, MN996528.1, MN996529.1, MN996530.1, MN996531.1, MT135041.1, MT135043.1, MT027063.1, and MT027062.1. The bolded ID represents the GenBank reference sequence. Then, the translated coding sequences of the four structural protein sequences were aligned and compared using a multiple sequence alignment (Clustal Omega, EMBL-EBI, United Kingdom). Of the 19 sequences, 15 were identical; however, single AA changes occurred in four N protein sequences: MN988713.1, N 194 S->X; MT135043.1, N 343 D->V; MT027063.1, N 194 S->L; MT027062.1, N 194 S->L. The resulting AA substitutions affected only two positions of N protein sequence (AA 194 and 343), neither of which occurred in epitopes that have been selected as targets for vaccine development.

Recent report (Feb.2021) established four different lineages by analyzing 45,494 complete SARS-CoV-2 genome sequences in the world. Most frequent circulating mutations from this report identified 11 missense amino acid mutations, one in S protein (D614G), three located in N protein (R203K with two different DNA substitutions and G204R), and further seven mutations in NSP2, NSP12, NSP13, ORF3a, and ORF8 (Wang et al., 2021). None of these amino acid positions were included in the nine 30-mers, supporting the proper selection of the conservative regions and intention to identify universal vaccine candidate peptides. Additionally, none of PolyPEPI-SCoV-2 peptides is affected by the presently, emerging mutant SARS-CoV-2 strains, except one single amino acid substitution: B.1.1.7 (UK, 17 mutations: delH69, V70, and Y144, substitutions in S: N501Y, A570D, D614G, P681H, T716I, S982A, D1118H; in N: D3L, S235F; and five mutant positions in ORF1ab), B.1.351 (South Africa, 10 mutations: amino acid substitutions in S: L18F, D80A, D215G, R246I, K417N, E484K, N501Y, A701V; in N: T205I, a single P71L change that affected one amino acid position in our peptide E1, and one non-affecting mutation in ORF1ab) or B.1.1.28.1 (Brazil, 16 mutations in S: L18F, T20N, P26S, D138Y, R190S, K417T, E484K, N501Y, H655Y, T1027I; in N: P80R, and five mutations in ORF1ab), or B.1.617 (India, “double mutant” with S protein substitutions L452R, E484Q, D614G), B.1.618 (India, “triple mutant” with S protein delH145-146, and substitutions L452R, E484Q, D614G) either^3^ (Rambaut et al., 2020; Thomson et al., 2021; O'Toole et al., 2021a,b; Tada et al., 2021). Further details on peptide selection are provided in the Results section and the resulting composition of the nine selected 30-mer peptides is shown in Table 1.

**Table 1:** PolyPEPI-SCoV-2 peptides and comprising PEPI frequencies within the *in silico* human cohort.

**SARS-CoV-2 fragment**	**ID**	**Peptide (30-mer)**	**Class I PEPI**	**Class II PEPI**	**B cell epitope in SARS (ref)**
S (35–64)	S2	GVYYPDKVFRSSVLH**STQDLFLPF****FSNVTW**	71%	94%	N/A
S (253–282)	S5	DSSSGWTAGAAAYYVG**YLQPRTFL****L**KYNEN	84%	97%	N/A
S (893–922)^†^	S9	ALQIP**FAMQMAYRF****N**GIGVTQNVLYENQKL	93%	99%	IgM, 50% (*n* = 4) (Guo et al., 2004)
N (36–65)^†^	N1	RSKQRRPQGLPN**NTASWFTAL****TQHGK**EDLK	36%	36%	IgG, 62% (*n* = 42) (He et al., 2004; Liu et al., 2006)
N (255–284)	N2	SKKPRQKRTAT**KAYNVTQAF****GRR**GPEQTQG	48%	22%	N/A
N (290–319)^†^	N3	EL*IRQGTDYKHWP**Q*IAQ**FAPSASAFF****GM**SR	54%	50%	IgG, 34% (*n* = 42) (He et al., 2004) IgG, IgM, 50% (*n* = 4) (Guo et al., 2004)
N (384–413)^†^	N4	QRQKKQQTVT*LLPAAD*LDD**FSKQLQQSM**SS	23%	36%	IgG, IgM, 95% (*n* = 42) (He et al., 2004) IgG, IgM, 75% (*n* = 4) (Guo et al., 2004)
M (93–122)	M1	LSYFIASF**RLFARTR****SM**WSFNPETNILLNV	90%	100%	N/A
E (45–74)	E1	NIVNVSLVKPSF**YVYSRVKNL****NS**SRVPDLL	46%	100%	N/A
**Combined frequency of PolyPEPI-SCoV-2 PEPIs**					
At least one peptide			100%	100%	N/A
At least two peptides			100%	100%	
At least three peptides			97%	100%	

*Bold: 9-mer HLA class I PEPI sequences; underlined: 15-mer HLA class II PEPI sequences within the PolyPEPI-SCoV-2 comprising 30-mer peptides. Percentages show the proportion of individuals from the model population with at least one HLA class I (CD8^+^ T cell specific) PEPI or at least one HLA class II (CD4^+^ T cell specific) PEPI. Peptides labeled*

† *contain experimentally confirmed B cell epitopes with antibody (Ig) responses found in convalescent patients with SARS. N/A, data not available*.

#### Cross-Reactivity With Human Coronavirus Strains

The sequence of PolyPEPI-SCoV-2 vaccine was compared with that of SARS-CoV, MERS-CoV and common (seasonal) human coronavirus strains to reveal possible cross-reactive regions. According to Centers for Disease Control and Prevention (CDC), common coronaviral infections in the human population are caused by four coronavirus groups: alpha coronavirus 229E and NL63, and beta coronavirus OC43 and HKU1. Pairwise alignment of the structural proteins was also performed using UniProt database with the following reference sequence IDs: 229E: P15423 (S), P15130 (N), P19741 (E), P15422 (M); NL63: Q6Q1S2 (S), Q6Q1R8 (N), Q6Q1S0 (E), Q6Q1R9 (M); OC43: P36334 (S), P33469 (N), Q04854 (E), Q01455 (M); HKU1 (Isolate N1): Q5MQD0 (S), Q5MQC6 (N), Q5MQC8 (E), Q5MQC7 (M) (Consortium, The UniProt, 2018). In addition, the coronavirus strains were aligned with the nine 30-mer peptides comprising the PolyPEPI-SCoV-2 vaccine. For the minimum requirement of an epitope, eight AA-long regions were screened (sliding window) as regions responsible for potential cross-reactivity. In addition, shorter (and longer) length matching consecutive peptide fragments were recorded and reported during the analysis.

#### No Cross-Reactivity With Human Protein Sequences

The selected immunogenic peptide candidates of PolyPEPI-SCoV-2 were analyzed by Basic Local Alignment Search (BLAST) analysis to identify any unwanted immunogenic regions in the vaccine that overlap with any proteins or peptides of the human proteome, available at blast.ncbi.nlm.nih.gov. All nine 30-mer peptide sequences were evaluated for homology with human proteins by comparing the sequences against the human protein database (taxid:9606). No cross-reactivity defined by at least eight consecutive amino acid match has been found between the PolyPEPI-SCoV-2 peptides and proteins in the human proteome, consequently, no related autoimmune reactions are expected due to sequence similarities.

#### Peptides and PolyPEPI-SCoV-2 Vaccine Preparation

The 9-mer (s2, s5, s9, n1, n2, n3, n4, e1, m1) and 30-mer (S2, S5, S7, N1, N2, N3, N4, E1, M1) peptides were manufactured by Intavis Peptide Services GmbH&Co. KG (Tübingen, Germany) and PEPScan (Lelystad, The Netherlands) using solid-phase peptide synthesis. Amino acid sequences are provided in Table 1 for both 9-mer test peptides (Table 1, bold) and the 30-mer vaccine peptides. Research grade PolyPEPI-SCoV-2 vaccine for the animal study was prepared by dissolving equal masses of the nine 30-mer peptides in DMSO (Sigma, Hungary) to achieve at a concentration of 1 mg/mL and then diluted with purified water to a final concentration of 0.2 mg/mL and stored frozen until use. Ready-to-inject vaccine preparations were prepared by emulsifying equal volumes of thawed peptide mix solution and Montanide ISA 51 VG adjuvant (Seppic, France) following the standard two-syringe protocol provided by the manufacturer.

### Epitope Prediction and Analysis

Prediction of ≥3HLA class I allele binding epitopes (PEPIs) for each individual was performed using an Immune Epitope Database (IEDB)-based epitope prediction method. The antigens were scanned with overlapping 9-mer to identify peptides that bind to the subject's HLA class I alleles. Selection parameters were validated with an in-house set of 427 HLA-epitope pairs that had been characterized experimentally by using direct binding assays (327 binding and 100 non-binding HLA-epitope pairs). Both specificity and sensitivity resulted in 93% for the prediction of true HLA allele-epitope pairs. HLA class II epitope predictions were performed by NetMHCpan (2.4) prediction algorithm for overlapping 15-mer peptides.

### Preclinical Animal Study Design

Thirty-six Hu-mice and 36 BALB/c mice received PolyPEPI-SCoV-2 vaccine (0.66 mg/kg/peptide in 200 μL solution; *n* = 18) or 20% DMSO/water (Sigma, Hungary and MilliQ purified water) emulsified in Montanide ISA 51 VG (Seppic, France) adjuvant (200 μL vehicle; *n* = 18) administered subcutaneously on days 0 and 14; the follow up period ended on day 28. Samples from days 14, 21, and 28 were analyzed (*n* = 6 per cohort). The studies were performed at the Transcure Bioservices facility (Archamps, France). The mice were monitored daily for unexpected signs of distress. Complete clinical scoring was performed weekly by monitoring coat (score 0–2), movement (0–3), activity (0–3), paleness (0–2), and bodyweight (0–3); a normal condition was scored 0.

All procedures described in this study have been reviewed and approved by the local ethic committee (CELEAG) and validated by the French Ministry of Research. Vaccination-induced T cell responses were assessed by *ex vivo* ELISpot and intracellular cytokine staining (ICS) assays of mice splenocytes (detailed below). Antibody responses were investigated by the measurement of total IgG in plasma samples (detailed below).

### ELISpot/FluoroSpot Assays

*Ex vivo* ELISpot assays for animal studies were performed as follows. IFN-γ-producing T cells were identified using 2 × 10^5^ splenocytes stimulated for 20 h/peptide (10 μg/ml, final concentration). Splenocytes were treated with 9-mer peptides (a pool of four N-specific peptides, N-pool (n1, n2, n3, n4), a pool of three S-specific peptides, S-pool (s2, s5, s9), an E protein-derived peptide, e1 or a M protein-derived peptide, m1) or with 30-mer peptides pooled the same way as 9-mers (N-pool comprising peptides N1, N2, N3, and N4), S-pool comprising peptides S2, S5, and S9, and individual peptides E1 and M1. ELISpot assays were performed using Human IFN-γ ELISpot PRO kit (ALP; ref 3321-4APT-2) from Mabtech for Hu-mice cohorts and Mouse IFN-γ ELISpot PRO kit (ALP; ref 3321-4APT-10) from Mabech for BALB/c mice cohorts, according to the manufacturer's instructions. Unstimulated (DMSO) assay control background spot forming unit (SFU) was subtracted from each data point and then the delta SFU (dSFU) was calculated. PMA/Ionomycin (Invitrogen) was used as a positive control.

*Ex vivo* FluoroSpot assays for convalescent donor testing were performed by Nexelis-IMXP (Belgium) as follows: IFN-γ/IL-2 FluoroSpot plates were blocked with RPMI-10% FBS, then peptides (5 μg/mL final concentration) or peptide pools (5 μg/mL per peptide final concentration) were added to the relevant wells. PBMCs of *N* = 17 convalescent donors and *N* = 4 healthy controls were retrieved from cryogenic storage and thawed in culture medium. Then, 200,000 PBMC cells/well were plated in triplicate (stimulation conditions) or 6-plicates (reference conditions) and incubated overnight at 37°C, 5% CO_2_ before development. Development of the FluoroSpot plates was performed according to the manufacturer's recommendations. After removing cells, detection antibodies diluted in PBS containing 0.1% BSA were added to the wells and the FluoroSpot plates were incubated for 2 h at room temperature. Before read-out using the Mabtech IRIS™ automated FluoroSpot reader, the FluoroSpot plates were emptied and dried at room temperature for 24 h protected from light. All data were acquired with a Mabtech IRIS™ reader and analyzed using Mabtech Apex TM software. Unstimulated (DMSO) negative control, CEF positive control (T cell epitopes derived from CMV, EBV and influenza, Mabtech, Sweden), and a commercial SARS-CoV-2 peptide pool (SARS-CoV-2 S N M O defined peptide pool (3622-1)—Mabtech, Sweden) were included as assay controls. *Ex vivo* FluoroSpot results were considered positive when the test result was higher than DMSO negative control after subtracting non-stimulated control (dSFU).

Enriched FluoroSpot assays for convalescent donor testing were performed by Nexelis-IMXP (Belgium) as follows: PBMCs were retrieved from cryogenic storage and thawed in culture medium. The PBMCs of *N* = 17 convalescent donors and *N* = 5 healthy controls were seeded at 4,000,000 cells/24-well in presence of the peptide pools (5 μg/ml per peptide) and incubated for 7 days at 37°C, 5% CO_2_. On days 1 and 4 of culture, the media were refreshed and supplemented with 5 ng/mL IL-7 or 5 ng/mL IL-7 and 4 ng/ml IL-2 (R&D Systems), respectively. After 7 days of culture, the PBMCs were harvested and rested for 16 h. The rested PBMCs were then counted using Trypan Blue Solution, 0.4% (VWR) and the Cellometer K2 Fluorescent Viability Cell Counter (Nexcelom), and seeded on the IFN-γ/Granzyme-B/TNF-α FluoroSpot plates (Mabtech) at 200,000 cells/well in RPMI 1640 with 10% Human Serum HI, 2 mM L-glutamin, 50 μg/ml gentamycin, and 100 μM β-ME into the relevant FluoroSpot wells containing peptide (5 μg/mL), or peptide pool (5 μg/mL per peptide), in triplicates. The FluoroSpot plates were incubated overnight at 37°C, 5% CO_2_ before development. All data were acquired with a Mabtech IRIS™ reader and analyzed using Mabtech Apex TM software. DMSO, medium only, a commercial COVID peptide pool (SARS-CoV-2 S N M O defined peptide pool [3622-1]—Mabtech), and CEF were included as assay controls at a concentration of 1 μg/ml. The positivity criterion was >1.5-fold the unstimulated control after subtracting the background (dSFU).

### Intracellular Cytokine Staining Assay

*Ex vivo* ICS assays for preclinical animal studies were performed as follows: splenocytes were removed from the ELISpot plates after 20 h of stimulation, transferred to a conventional 96-well flat bottom plate, and incubated for 4 h with BD GolgiStop™ according to the manufacturer's recommendations. Flow-cytometry was performed using a BD Cytofix/Cytoperm Plus Kit with BD GolgiStop™ protein transport inhibitor (containing monensin; Cat. No. 554715), following the manufacturer's instructions. Flow cytometry analysis and cytokine profile determination were performed on an Attune NxT Flow cytometer (Life Technologies). A total of 2 × 10^5^ cells were analyzed, gated for CD45^+^, CD3^+^, CD4^+^, or CD8^+^ T cells. Counts below 25 were evaluated as 0. Spot counts ≥ 25 were background corrected by subtracting unstimulated (DMSO) control. PMA/Ionomycin (Invitrogen) was used as a positive control. As an assay control, Mann-Whitney test was used to compare negative control (unstimulated) and positive control (PMA/ionomycin) for each cytokine. When a statistical difference between controls was determined, the values corresponding to the other stimulation conditions were analyzed. The following stains were used for Hu-mice cohorts: MAb11 502932 (Biolegend), MP4-25D2 500836 (Biolegend), 4S.B3 502536 (Biolegend), HI30 304044 (Biolegend), SK7 344842 (Biolegend), JES6-5H4 503806 (Biolegend), VIT4 130-113-218 (Miltenyi), JES1-39D10 500904 (Biolegend), SK1 344744 (Biolegend), JES10-5A2 501914 (Biolegend), JES3-19F1 554707 (BD), and NA 564997 (BD). The following stains were used for BALB/c mice cohorts: 11B11 562915 (BD), MP6-XT22 506339 (Biolegend), XMG1.2 505840 (Biolegend), 30-F11 103151 (Biolegend), 145-2C11 100355 (Biolegend), JES6-5H4 503806 (Biolegend), GK1.5 100762 (Biolegend), JES1-39D10 500904 (Biolegend), 53-6.7 100762 (Biolegend), eBio13A 25-7133-82 (Thermo Scientific), JESS-16E3 505010 (Biolegend), and NA 564997 (BD).

*Ex vivo* ICS assays for convalescent donor testing were performed by Nexelis-IMXP (Belgium). Briefly, after thawing 200,000 PBMC cells/well, PBMCs were seeded in sterile round-bottom 96-well plates in RPMI total with 10% human serum HI, 2 mM L-glutamine, 50 μg/mL gentamycin, and 100 μM 2-ME in the presence of peptides (5 μg/mL) or peptide pool (5 μg/mL per peptide). After a 2-h incubation, BD GolgiPlug™ (BD Biosciences) was added to the 96-well plates at a concentration of 1 μl/ml in culture medium. After a 10-h incubation, plates were centrifuged (800 g, 3 min, 8°C) and incubated for 10 min at 37°C and Zombie NIR Viability dye (Biolegend) was added to each well. Plates were incubated at room temperature for 15 min, shielded from the light. After incubation, PBS/0.1% BSA was added per well and the plates were centrifuged (800 g, 3 min, 8°C). Thereafter, cells were incubated in FcR blocking reagent at 4°C for 5 min, and then staining mixture (containing anti-CD3, Biolegend, anti-CD4, and anti-CD8 antibodies; BD Biosciences) was added to each well. After 30 min of incubation at 4°C, washing, and centrifugation (800 g, 3 min, 8°C), cells were permeabilized and fixed according to the manufacturer's recommendations (BD Biosciences). After fixation, cytokine staining mixture (containing anti-IFN-γ, anti-IL-2, anti-IL-4, anti-IL-10 and anti-TNF-α antibodies, Biolegend) was added to each well. Plates were incubated at 4°C for 30 min and then washed twice before acquisition. All flow cytometry data were acquired with LSRFortessa™ X-20 and analyzed using FlowJo V10 software. DMSO negative control was subtracted from each data point obtained using test peptides or pools.

### Antibody ELISA

ELISAs for mouse studies were performed for the quantitative measurement of total mouse IgG production in plasma samples using IgG (Total) Mouse Uncoated ELISA Kit (Invitrogen, #88-50400-22) for BALB/c cohorts and IgG (Total) Human Uncoated ELISA Kit (Invitrogen, #88-50550-22) for Hu-mice cohorts according to the manufacturer's instructions. Analyses were performed using samples harvested at days 14, 21, and 28 (*n* = 6 per group per time point). Absorbance were read on an Epoch Microplate Reader (Biotech) and analyzed using Gen5 software.

Euroimmune ELISA assays for convalescent donors were performed to determine S1-specific IgG levels via the independent medical research center, The Netherlands. The Anti-SARS-CoV-2 ELISA plates are coated with recombinant S-1 structural protein from SARS-CoV-2 to which antibodies against SARS-CoV-2 bind. This antigen was selected for its relatively low homology to other coronaviruses, notably SARS-CoV. The immunoassay was performed according to the manufacturer's instructions.

ELISAs were performed by Mikromikomed Kft (Budapest, Hungary) using a DiaPro COVID-19 IgM Enzyme Immunoassay for the determination of IgM antibodies to COVID-19 in human serum and plasma, DiaPro COVID-19 IgG Enzyme Immunoassay for the determination of IgG antibodies to COVID-19 in human serum and plasma, and DiaPro COVID-19 IgA Enzyme Immunoassay for the determination of IgA antibodies to COVID-19 in human serum and plasma, according to the manufacturer's instructions (Dia.Pro Diagnostic Bioprobes S.r.l., Italy). For the determination of N-specific antibodies, Roche Elecsys® Anti-SARS-CoV-2 Immunoassay for the qualitative detection of antibodies (including IgG) against SARS-CoV-2 was used with a COBAS e411 analyzer (disk system; ROCHE, Switzerland) according to the manufacturer's instructions.

(Vero C1008 (ATCC No." should be replaced with "(Vero C1008, ATCC No.

### Pseudoparticle Neutralization Assay

Neutralizing antibodies in mice sera were assessed using a cell-based Pseudoparticle Neutralization Assay. Vero E6 cells expressing the ACE-2 receptor (Vero C1008 ATCC No. CRL-1586, US), were seeded at 20 000 cells/well to reach a cell confluence of 80%. Serum samples and controls (pool of human convalescent serum, internally produced) were diluted in duplicates in cell growth media at a starting dilution of 1/25 (for samples) or 1/100 (for controls), followed by a serial dilution (2-fold dilutions, five times). In parallel, SARS-CoV-2 pseudovirus (prepared by Nexelis, using Kerafast system), with Spike from Wuhan Strain (minus 19 C-terminal amino acids) was diluted as to reach the desired concentration (according to pre-determined TU/mL). Pseudovirus was then added to diluted sera samples and pre-incubated for 1 h at 37°C with CO2. The mixture was then added to the pre-seeded Vero E6 cell layers and plates were incubated for 18–24 h at 37°C with 5% CO_2_. Following incubation and removal of media, ONE-Glo EX Luciferase Assay Substrate, Promega, Cat. E8110) was added to cells and incubated for 3 min at room temperature with shaking. Luminescence was measured using a SpectraMax i3x microplate reader and SoftMax Pro v6.5.1 (Molecular Devices). Luminescence results for each dilution were used to generate a titration curve using a 4-parameter logistic regression (4PL) using Microsoft Excel (for Microsoft Office 365). The titer was defined as the reciprocal dilution of the sample for which the luminescence is equal to a pre-determined cut-point of 50, corresponding to 50% neutralization. This cut-point was established using linear regression using 50% flanking points.

### Statistical Analysis

Significance was compared between and among groups using *t*-tests, Mann-Whitney tests, or Permutation statistics using Montecarlo simulations, as appropriate. *p* < 0.05 was considered significant. Pearson's test and/or Spearman's test was performed to assess correlations. The correlation was considered strong if *R* > 0.7, moderate, if 0.5 < *R* ≤ 0.7 and weak, if 0.3 < *R* ≤ 0.5. Dependent variables were determined using Fisher Exact test for a 2 × 2 contingency table.

## Results

### Tailoring PolyPEPI-SCoV-2 to Individuals' Genetic Profile

During the design of PolyPEPI-SCoV-2, we used the HLA genotype data of subjects in the *in silico* human cohort (Model Population) to determine the most immunogenic peptides (i.e., HLA class I PEPI hotspots, 9-mers) of the four selected SARS-CoV-2 structural proteins aimed to induce CD8^+^ T cell responses. The sequences of the identified 9-mer PEPI hotspots were then extended to 30-mers based on the viral protein sequences to maximize the number of HLA class II binding PEPIs (15-mers) aimed to induce CD4^+^ T cell responses as detailed below.

**Figure 1:**
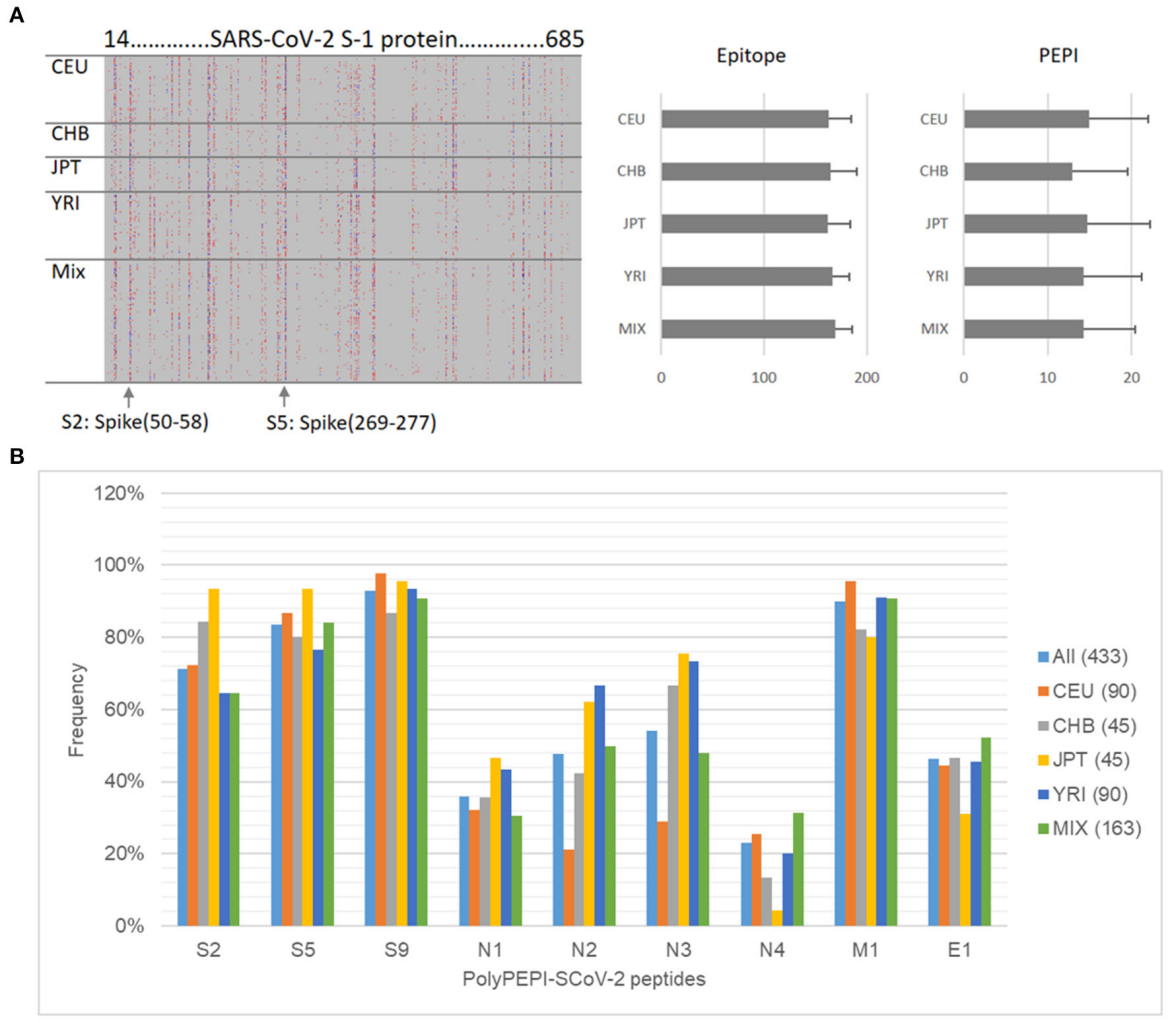
Design of PolyPEPI-SCoV-2. **(A)** Hotspot analysis of SARS-CoV-2 Spike-1 protein in the ethnically diverse *in silico* human cohort. Analysis was performed by predicting ≥3 HLA allele binding personal epitopes (PEPIs) for each subject. Left panel: Each row along the vertical axis represents one subject in the model population, while the horizontal axis represents the SARS-CoV-2 S-1 protein sequence. Vertical bands represent the most frequent PEPIs, i.e., the dominant immunogenic protein regions (hotspots). Colors represent the number of epitopes restricted to a subject's: red, 3; green/blue, 4; black, >5 HLA class I allele. Right panel, average number of epitopes/PEPIs found for subjects of different ethnicities. **(B)** Heterogeneity of peptide frequencies in different ethnic groups. CEU, Central European; CHB, Chinese; JPT, Japanese; YRI, African; Mix, mixed ethnicity subjects.

First, we performed epitope predictions for each subject in the *in silico* human cohorts for each of their HLA class I and class II alleles (six HLA class I and class II alleles) for the AA sequence of the conserved regions of 19 known SARS-CoV-2 viral proteins using 9-mer (HLA class I) and 15-mer (HLA class II) frames, respectively (Figure 1A; section Materials and Methods). Then, we selected the epitopes restricted to multiple (≥3) autologous HLA alleles (PEPIs) to account for the most abundantly presented epitopes. We identified several HLA-restricted epitopes (average, 166 epitopes only for S1 protein) for each person. In contrast, PEPIs are represented at much lower level in all ethnicities (average, 14 multi-HLA binding epitopes, Figure 1A). Of note, we did not observe any difference in SARS-CoV-2 S1-protein specific epitope generation capacity of individuals with different ethnicities based on their complete HLA genotype, which does not seem to explain the higher infection and mortality rates observed in BAME. Instead, we observed heterogeneity in the frequency of the shared PEPIs in the different ethnic groups, especially for protein N, having high impact on the design of a potential global vaccine (Figure 1B). Combination of targets with different frequencies inside- and between ethnic groups into a vaccine candidate with high global coverage is feasible only by performing “*in silico* clinical trials” in large populations of real subjects.

Therefore, to maximize multi-antigenic immune responses at both the individual and population/ethnicity levels, and also considering the chemical and manufacturability properties of the peptides, we selected a total of nine 30-mer peptides from four structural proteins of SARS-CoV-2: three peptides from spike (S), four peptides from nucleoprotein (N), and one peptide from each matrix (M) and envelope (E). No peptides were included from the receptor-binding domain (RBD) of S protein. Overall, each member of the Model Population had HLA class I PEPIs for at least two of the nine peptides, and 97% had at least three (Table 1). Each subject had multiple class II PEPIs for the vaccine peptides (Table 1).

We identified experimentally confirmed linear B cell epitopes derived from SARS-CoV, with 100% sequence identity to the relevant SARS-CoV-2 antigen, to account for the potential B cell production capacity of the long peptides (Ahmed et al., 2020). Three overlapping epitopes located in N protein- and one epitope in S protein-derived peptides of PolyPEPI-SCoV-2 vaccine were reactive with the sera of convalescent patients with severe acute respiratory syndrome (SARS). This suggests that the above antigenic sites on the S and N protein are important in eliciting humoral immune response against SARS-CoV and likely against SARS-CoV-2, in humans.

None of the peptides involved in PolyPEPI-SCoV-2 composition are cross-reactive with the human genome at minimal epitope level, as assessed by BLAST analysis (see section Methods). As expected, PolyPEPI-SCoV-2 contains several (eight out of nine) peptides that are cross-reactive with SARS-CoV due to high sequence homology between SARS-CoV-2 and SARS-CoV. Sequence similarity is low between the PolyPEPI-SCoV-2 peptides and common (seasonal) coronavirus strains belonging to alpha coronavirus (229E and NL63), beta coronavirus (OC43, HKU1) and MERS. Therefore, cross-reactivity between the vaccine and prior coronavirus-infected individuals remains low and might involve only the M1 peptide of the vaccine (See section Materials and Methods; Supplementary Table 5). However, none of the peptides involved in the PolyPEPI-SCoV-2 vaccine composition is affected by the emergent SARS-CoV-2 variants and mutations known to date (See Materials and Methods for the analysis).

### PolyPEPI-SCoV-2-Specific T Cell Responses Detected in COVID-19 Convalescent Donors

Next, we aimed to demonstrate that shared PEPIs identified for the *in silico* cohort are also present in the T cell repertoire of natural SARS-CoV-2 infection by investigating vaccine-specific T cells circulating in the blood of COVID-19 convalescent donors (donor information are reported in Supplementary Tables 1, 2).

**Figure 2:**
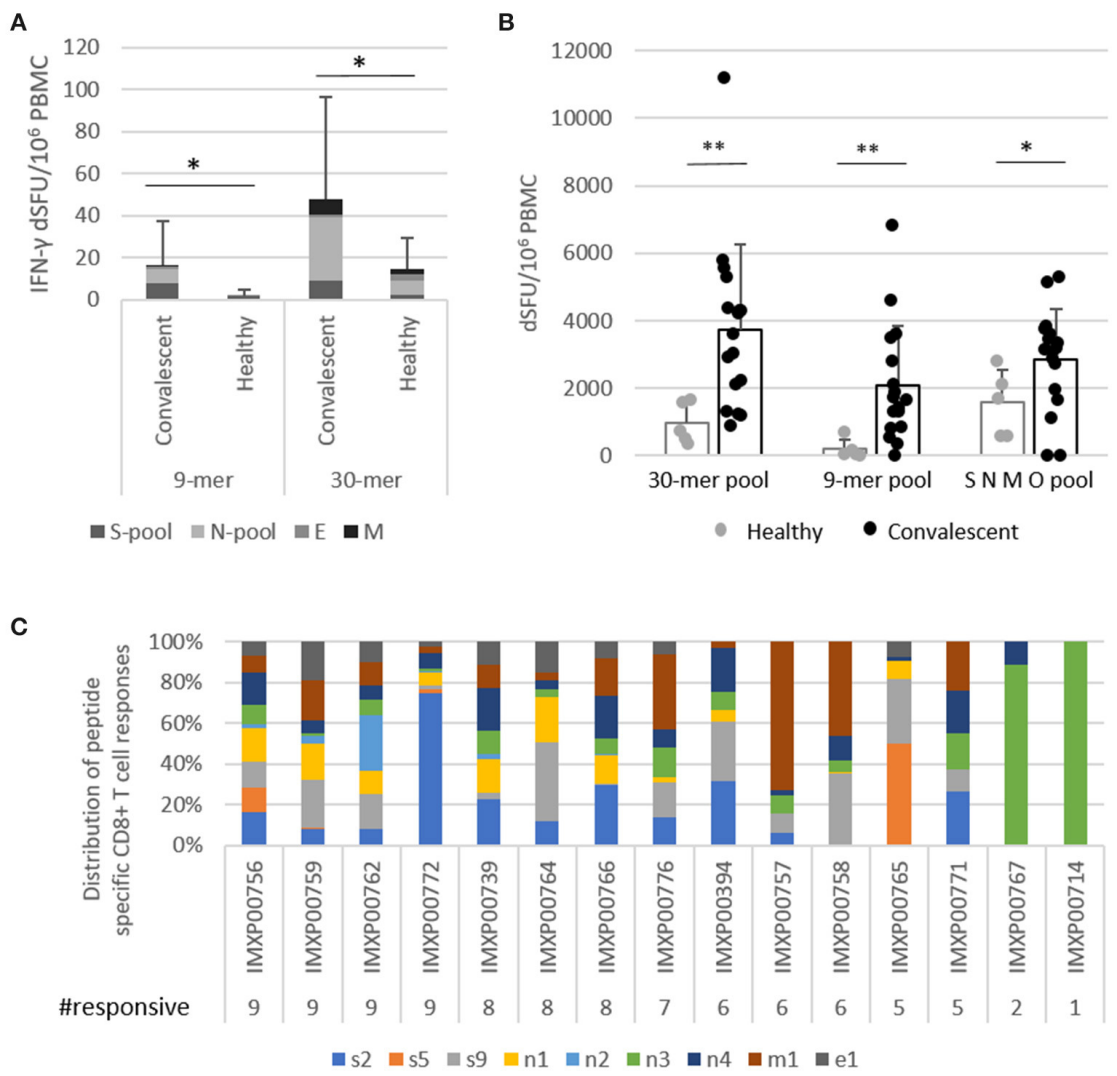
PolyPEPI-SCoV-2-specific T cells detected for COVID-19 convalescent donors. **(A)** Highly specific vaccine-derived 9-mer peptide-reactive CD8^+^ T cells and 30-mer peptide-reactive CD4^+^ T cells detected by *ex vivo* FluoroSpot assay. Test conditions: S-pool contains the three peptides derived from S protein; N-pool contains the four peptides derived from N protein; M and E are the pepti/des derived from M and E proteins, respectively, in both the 9-mer and 30-mer pools. **(B)** IFN-γ producing T cells activated with 30-mer peptides in one pool, 9-mer peptides in one pool, and a commercial SNMO peptide pool detected using enriched FluoroSpot assay. **(C)** IFN-γ producing CD8^+^ T cells activated by individual 9-mer peptides corresponding to each of the 30-mer peptides with the same name (Table 1 bold), detected using enriched FluoroSpot assay. dSFU, delta spot forming units, calculated as background corrected spot counts per 10^6^ PBMC. Significance was calculated using Permutation statistics with Montecarlo simulations; *p < 0.05, **p < 0.00005.

First, the reactivity of vaccine peptides with convalescent immune components was investigated in 17 convalescent and four healthy donors using *ex vivo* FluoroSpot assay which can detect rapidly activating, effector phase T cell responses. Vaccine-reactive CD4^+^ T cells were detected using the nine 30-mer vaccine peptides grouped in four pools according to their source protein: S, N, M, and E peptides. CD8^+^ T cell responses were measured using the 9-mer test peptides as well corresponding to the shared HLA class I PEPIs defined for each of the nine vaccine peptides, grouped into four pools (s, n, m, and e peptides; Table 1, bold). Significant amount of vaccine-reactive, IFN-γ-expressing T cells were detected with both 30-mer (average dSFU: 48.1) and 9-mer peptides (average dSFU: 16.5) compared with healthy subjects (Figure 2A). Detailed analysis of the four protein-specific peptide pools revealed that three out of the 17 donors reacted to all four structural proteins with the 30-mer vaccine peptides; 82% of donors reacted to two proteins and 59% to three proteins. Notably, highly specific 9-mer-detected CD8^+^ T cell responses could be also identified against at least one of four proteins in all 17 donors and against at least two proteins in 53% (Supplementary Table 6).

As determined by ICS assay, stimulation with 9-mer test peptides resulted in an average T cell make up of 83% CD8^+^ T cells, and 17% CD4^+^ T cells (Supplementary Figures 2A,B). The 30-mer peptides reacted with both CD4^+^ and CD8^+^ T cells in average ratio of 50:50 (Supplementary Figure 2B). Functionality testing of the T cells revealed that CD8^+^ T cells primarily produced IFN-γ, TNF-α, and IL-2 (with small amounts of IL-4 and IL-10), while CD4^+^ T cells were positive for mainly IL-2 and IFN-γ. Recall responses demonstrated clear Th1 cytokine characteristics; Th2 responses were not present in the recall response detected with 30-mer vaccine peptides (Supplementary Figure 2C).

Next, we determined whether the *ex vivo* detected T cells could also expand *in vitro* in the presence of vaccine peptides. Using enriched FluoroSpot, significant numbers of vaccine-reactive, IFN-γ-expressing T cells were detected with both 30-mer (average dSFU = 3,746) and 9-mer (average dSFU = 2,088) peptide pools compared with healthy subjects (Figure 2B). The intensity of the PolyPEPI-SCoV-2-derived T cell responses (30-mer pool) were also evaluated relative to the responses detected with a commercial, large SARS-CoV-2 peptide pool (SMNO) containing 47 long peptides derived from both structural (S, M, N) and non-structural (open reading frame ORF-3a and 7a) proteins. Interestingly, the magnitude of T cell responses were similar for the two peptide pools despite of the difference in their size, suggesting more prevalent responses for our peptide mix. In addition, the vaccine pool was favored by the COVID-19 donors, while healthy donors preferred the commercial peptide pool, confirming improved specificity of PolyPEPI-SCoV-2 peptides to SARS-CoV-2, in conformance with the result of cross-reactivity analysis with common coronavirus strains (Figure 2B).

To confirm and further delineate the multi-specificity of the PolyPEPI-SCoV-2-specific T cell responses of COVID-19 recovered individuals, we defined the distinctive peptides targeted by their T cells. We first deconvoluted the peptide pools and tested the CD8^+^ T cell responses specific to each of the 9-mer HLA class I PEPIs corresponding to each vaccine peptide using *in vitro* expansion (Figure 2C; Supplementary Figure 3). Analysis revealed that each 9-mer peptide was recognized by several subjects; the highest recognition rate in COVID-19 convalescent donors was observed for n4 and n3 (93%), s9 (87%), s2, n1, m1 (80%), e1 (60%), s5, n2 (40%) (Figure 2C). Detailed analysis of the nine peptide-specific CD8^+^ T cell responses revealed that 100% of COVID-19-recovered subjects had PolyPEPI-SCoV-2-specific T cells reactivated with at least one peptide, 93% with more than two, 87% with more than five, and 27% had T cell pools specific to all nine vaccine peptides. At the protein level, 87% of subjects had T cells against multiple (three) proteins and eight out of the 15 measured donors (53%) reacted to all four targeted viral proteins (Figure 2C). These data confirm that PolyPEPI-SCoV-2-peptides are dominant for an individual and shared between COVID-19 subjects. Convalescents' T cells recognizing PolyPEPI-SCoV-2-specific 9-mer peptides were fully functional, expressing IFN-γ and/or TNF-α and/or Granzyme-B (Supplementary Figure 4). For our cohort of convalescent subjects, the breadth and magnitude of vaccine-specific T cell responses were independent of time from symptom onset, suggesting that these T cells are persistent (for at least 5 months) (Supplementary Figure 5).

As an external validation, we determined the frequency of PolyPEPI-SCoV-2-specific T cell responses in a second convalescent cohort reported by Tarke et al. Uniquely, this study reports individual T cell response data to predicted MHC I and II-epitope pairs. Eight of our nine peptides (identical or overlapping sequences with at least eight amino acids) were tested for 42 convalescent subjects, with average 4.5 out of eight peptides being tested per subject.

We found that each of the eight peptides (100%) were shared between at least three subjects tested, peptide N3 was found for 13/27 (48%) of subjects (Supplementary Figure 6). Furthermore, 26/42 (62%) of subjects had T cell responses specific for at least one, 14/42 (33%) for at least two and 8/42 (19%) for at least three PolyPEPI-SCoV-2 peptides (Supplementary Figure 6). These data confirm the immunoprevalent nature of our peptides in an independent cohort of convalescents using an *ex vivo* T cell receptor dependent Activation Induced Marker (AIM) assay.

### Correlation Between PolyPEPI-SCoV-2-Reactive T Cells and SARS-CoV-2-Specific Antibody Responses

T cell-dependent B cell activation is required for antibody production. For each subject, different levels of antibody responses were detected against both S and N antigens of SARS-CoV-2 determined using different commercial kits (Table 1). All subjects tested positive with Euroimmune ELISA against viral S1 subunit (IgG-S1) and a Roche kit to measure N-related antibodies (IgG-N). All subjects tested positive for DiaPro IgG and IgM (except two donors), 7/17 for DiaPro IgA detecting mixed S1 and N protein-specific antibody responses (Supplementary Table 1).

**Figure 3:**
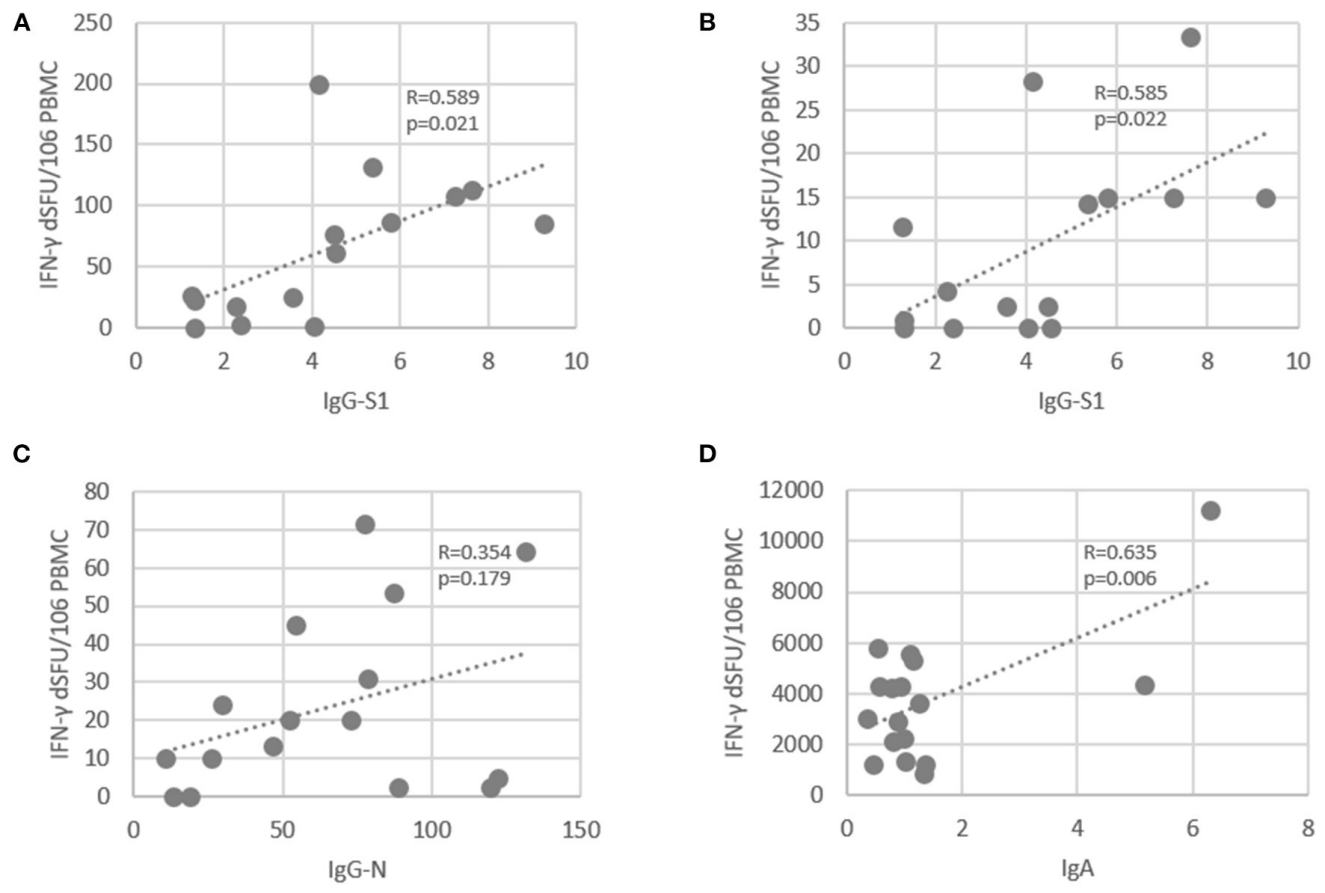
Correlation between SARS-CoV-2-specific antibody levels and PolyPEPI-SCoV-2-specific IFN-γ-producing CD4^+^ T cells in COVID-19 convalescent individuals. **(A)** T cell responses reactive to 30-mer pool of PolyPEPI-SCoV-2 peptides were plotted against the IgG-S1 (Euroimmune). **(B)** Average T cell responses reactive to S1 protein subunit-derived 30-mer peptides (S2 and S5) was plotted against IgG-S1 (Euroimmune). **(C)** T cell responses reactive to 30-mer N peptide pool comprising N1, N2, N3, and N4 was plotted against total IgG-N measured with Roche Elecsys® assay. **(D)** T cell responses reactive to 30-mer pool of PolyPEPI-SCoV-2 peptides were plotted against the IgA antibody amounts measured by DiaPro IgA ELISA assay. R: Pearson correlation coefficient.

We next evaluated the correlation between PolyPEPI-SCoV-2-specific CD4^+^ T cell reactivities and antibody responses (Figure 3). The total amount of PolyPEPI-SCoV-2-reactive CD4^+^ T cells correlated with IgG-S1 (*R* = 0.59, *p* = 0.02, Figure 3A). Next, the subset of CD4^+^ T cells reactive to specific S1 protein subunit-derived peptides of the PolyPEPI-SCoV-2 vaccine (S2 and S5) were analyzed and the correlation was similar (*R* = 0.585, *p* = 0.02, Figure 3B). T cell responses detected with N protein derived PolyPEPI-SCoV-2 peptides (N1, N2, N3, and N4) presented a weak but not significant correlation with IgG-N (Figure 3C). These data suggest a link between PolyPEPI-SCoV-2-specific CD4^+^ T cell responses and subsequent IgG production for COVID-19 convalescent donors. Interestingly, IgA production correlated with PolyPEPI-SCoV-2-specific memory CD4^+^ T cell responses (*R* = 0.63, *p* = 0.006, Figure 3D, although Spearman test did not confirm the correlation). T cell responses reactive to the SMNO peptide pool exhibited no correlation with any of the antibody subsets. This suggests that not all CD4^+^ T cells contributed to B cell responses, consequently to IgG production.

### Correlation Between Multiple Autologous Allele-Binding Epitopes (PEPIs) and CD8^+^ T Cell Responses

We investigated the HLA-binding capacity of the immunogenic peptides detected for each subject.

**Figure 4:**
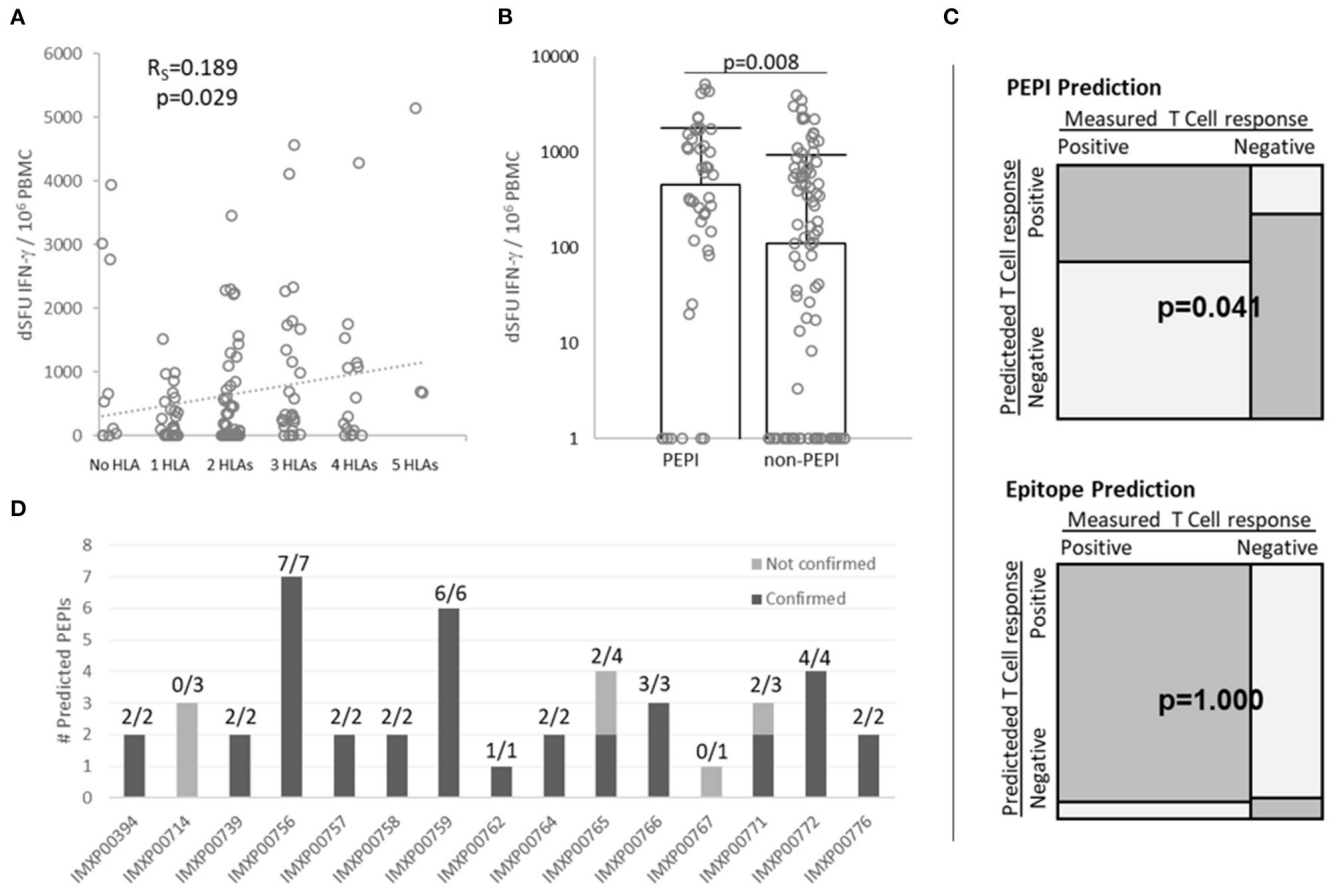
Correlation between multiple autologous HLA allele-binding epitopes and PolyPEPI-SCoV-2-specific IFN-γ-producing CD8^+^ T cell responses in COVID-19 convalescent individuals. **(A)** Correlation between multiple autologous HLA allele-binding epitopes and magnitude of T cell responses. R_s_, Spearman coefficient (confirmed by Pearson correlation analysis, too) **(B)** Magnitude of CD8+ T cell responses detected for PEPIs (binding ≥ 3 autologous HLA class I alleles) and for non-PEPIs (binding <3 autologous HLA class I alleles) by enriched FluoroSpot assay, (*p* = 0.008, *t*-test). Median and individual data for each subject are presented, *n* = 15 **(C)** Variable dependency analysis using 2 × 2 contingency table and Fisher Exact test. **(D)** Confirmation of Personal Epitopes (PEPIs) by IFN-γ producing CD8^+^ T cells for each subject [Positive Predictive Value (PPV) = True positive/Total predicted = 37/44 (84%)]. dSFU, delta spot forming units calculated as non-stimulated background corrected spot counts per 10^6^ PBMC; PBMC, peripheral blood mononuclear cells.

First we determined the complete HLA class I genotype for each subject and then predicted the number of autologous HLA alleles that could bind to each of the nine shared 9-mer peptides used in the FluoroSpot assay. Then we matched the predicted HLA-binding epitopes to the CD8^+^ T cell responses measured for each peptide in each patient (total 15 × 9 = 135 data points, Supplementary Figure 7). The magnitude of CD8^+^ T cell responses tended to correlate with epitopes restricted to multiple autologous HLA alleles (*R*_*S*_ = 0.188, *p* = 0.028, Figure 4A). In addition, we observed that the magnitude of CD8^+^ T cell responses generated by PEPIs (HLA ≥3) (median dSFU = 458) was significantly higher than those generated by non-PEPIs (HLA < 3) (median dSFU = 110), (*p* = 0.008) (Figure 4B).

Across the 135 data points there were 98 positive responses and 37 negative responses recorded. Among the 98 positive responses 37 were generated by PEPIs, while among the 37 negatives only seven were PEPIs, the others were epitopes restricted to <3 autologous HLA alleles (Supplementary Figure 7). Overall, the 2 × 2 contingency table revealed association of T cell responses with PEPIs (*p* = 0.041, Fisher Exact) but not with HLA-restricted epitopes (*p* = 1.000, Fisher Exact) (Figure 4C). For each subject between one and seven peptides out of nine proved to be PEPIs. Among the predicted PEPIs, 37/44 (84%) were confirmed by IFN-γ FluoroSpot assay to generate specific T cell responses in the given subject (Figure 4D; Supplementary Figure 7).

These data demonstrate that subjects' complete HLA-genotype influence their CD8^+^ T cell responses and multiple autologous allele-binding capacity is a key feature of immunogenic epitopes. PEPIs in general underestimated the subject's overall T cell repertoire, however they precisely predicted subjects' PEPI-specific CD8^+^T cell responses.

### Predicted Immunogenicity in Different Ethnicities

**Figure 5:**
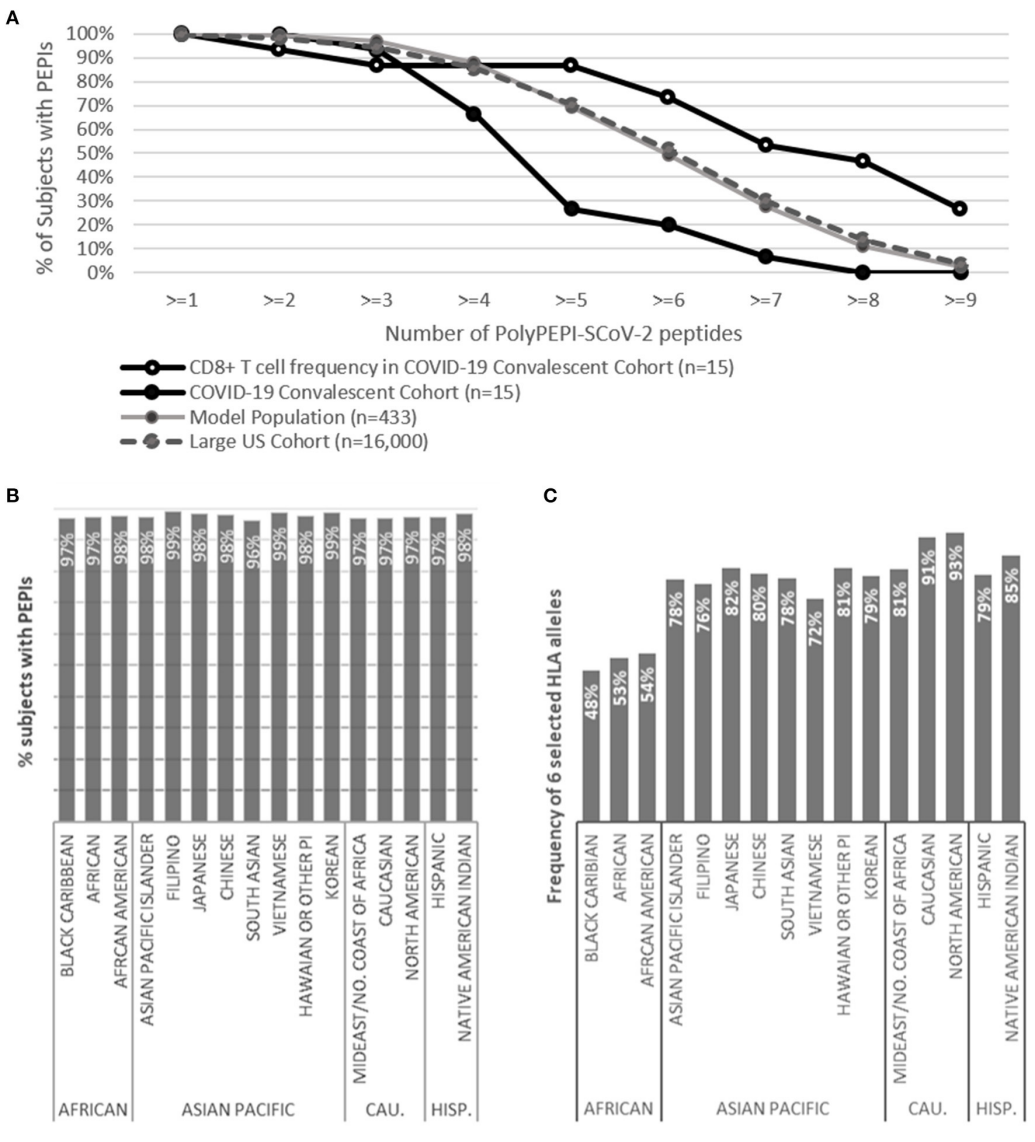
Predicted global coverage in a large population with different ethnicities. **(A)** Proportion of subjects having predicted HLA class I PEPIs against PolyPEPI-SCoV-2 peptides in different cohorts and the frequency of experimentally measured CD8^+^ T cell responses in the COVID-19 convalescent cohort (*n* = 15), obtained by FluoroSpot assay. **(B)** Proportion of subjects having both HLA class I and class II PEPIs against at least two peptides in the PolyPEPI-SCoV-2 vaccine. **(C)** Theoretical global coverage estimated based on the frequency of six prevalent HLA alleles (A*02:01, A*01:01, A*03:01, A*11:01, A*24:02, and B*07:02), as proposed by Ferretti et al. (2020). CAU., Caucasian; HISP., Hispanic; *n* = 16,000.

Since the T cell responses detected in convalescents validated our hypothesis that PEPIs determined for an individual's HLA genotype generate CD8^+^ T cell responses with high predictive value, we used this knowledge to determine the scalability of our approach and estimate the global coverage of our vaccine candidate. As expected, the measured peptide-specific CD8^+^ T cell frequencies obtained in the convalescent population were in good agreement with their predicted PEPI frequencies and also with the frequency of shared PEPIs of the Model Population (*n* = 433) cohort used for the design (100% for at least one peptide for both predicted PEPIs and measured CD8^+^ T cell frequencies; 93% measured T cell response vs. 100% predicted for at least two peptides) (Figure 5A; Table 1). The polypeptide-specific T cell responses were however underestimated by both the individual HLA-genotypes and the Model Population compared to measured T cell responses.

To estimate the scalability of our *in silico* model, we determined the PEPI frequencies for a large cohort of 16,000 HLA-genotyped subjects distributed among 16 different ethnic groups obtained from a US bone marrow donor database. The ethnic groups covered in this cohort are representative for the composition of the global population and involves 99.8% of the alleles cataloged in the CIWD database for > 8 million human subjects globally (compared to 97.4% in the Model Population) (see section Methods) (Hurley et al., 2020). The CIWD database contains frequent alleles (documented for ≥ 5 subjects) as well as rare alleles (documented for <5 subjects). The PEPI frequencies obtained for our Model Population (*n* = 433) and this large US cohort (*n* = 16,000) were in perfect alignment, suggesting high global coverage ensured by the high number of frequent alleles covered in the Model Population and an overall low impact of the rare alleles found in the individuals' HLA-genotype (Figure 5A; Supplementary Figures 8A–C). In the large US cohort, most subjects had a broad repertoire of predicted PEPIs that based on the above findings will likely be transformed to multiple virus-specific memory CD8^+^ T cell clones: 98% of subjects were predicted to have PEPIs against at least two vaccine peptides, and 95, 86, and 70% against three, four, and five peptides, respectively (Figure 5A).

*In silico* testing revealed that 96–99% of subjects in each ethnic group will likely mount robust cellular responses, with both CD8^+^ and CD4^+^ T cell responses against at least two peptides in the vaccine (Figure 5B). This predicted high response rate was also true for the ethnicities reported to have worse clinical outcomes from COVID-19 (Black, Asian) (Pan et al., 2020). Based on these data, we expect that the vaccine will provide global coverage, independent of ethnicity and geographic location.

We also used this cohort (and comprising ethnic groups) to assess theoretical global coverage as proposed by others, by filtering the sub-populations having at least one of the six prevalent HLA class I alleles considered to cover 95% of the global population (Maiers et al., 2007; Gonzalez-Galarza et al., 2019; Ferretti et al., 2020). Using this approach, we observed significant heterogeneity at the ethnicity level. While we confirmed that the selected six HLA alleles are prevalent in the Caucasian and North American cohorts (91–93%), the frequency of these alleles was lower in all other ethnic groups, especially in African populations (48–54%) (Figure 5C). We concluded that the proposed prevalent HLA allele set may cover the HLA frequency in an ethnically weighted global population, but epitope selection for vaccination purposes based only on these alleles would discriminate some etnnicities. Therefore, we propose using a representative model population that is sensitive to the heterogeneities in the human race and that allows selecting PEPIs shared among individuals across ethnicities.

### PolyPEPI-SCoV-2 Vaccine Candidate Induced Broad T Cell Responses in Two Animal Models

Preclinical immunogenicity testing of PolyPEPI-SCoV-2 was performed to measure the induced immune responses after one and two vaccine doses that were administered 2 weeks apart (days 0 and 14) in BALB/c and Hu-mouse models. After immunizations, no mice presented any clinical score at day 14, 21 or 28 (score 0, representing no deviation from normal), suggesting the absence of any side effects or immune aversion (Supplementary Tables 7A,B). In addition, the necropsies performed by macroscopic observation at each timepoint did not reveal any visible organ alteration in spleen, liver, kidneys, stomach and intestine (Supplementary Table 7C). Repeated vaccine administration was also well-tolerated, and no signs of immune toxicity or other systemic adverse events were detected. Together, these data strongly suggest that PolyPEPI-SCoV-2 was safe in mice.

Vaccine-induced IFN-γ producing T cells were measured after the first dose at day 14 and after the second dose at days 21 and 28. Vaccine-induced T cells were detected using the nine 30-mer vaccine peptides grouped in four pools according to their source protein: S, N, M, and E, to assess for the CD4^+^ and CD8^+^ T cell responses. CD8^+^ T cell responses were also specifically measured using the short 9-mer test peptides corresponding to the shared HLA class I PEPIs defined above for each of the nine vaccine peptides, in four pools (s, n, m, and e peptides; Table 1 bold).

**Figure 6:**
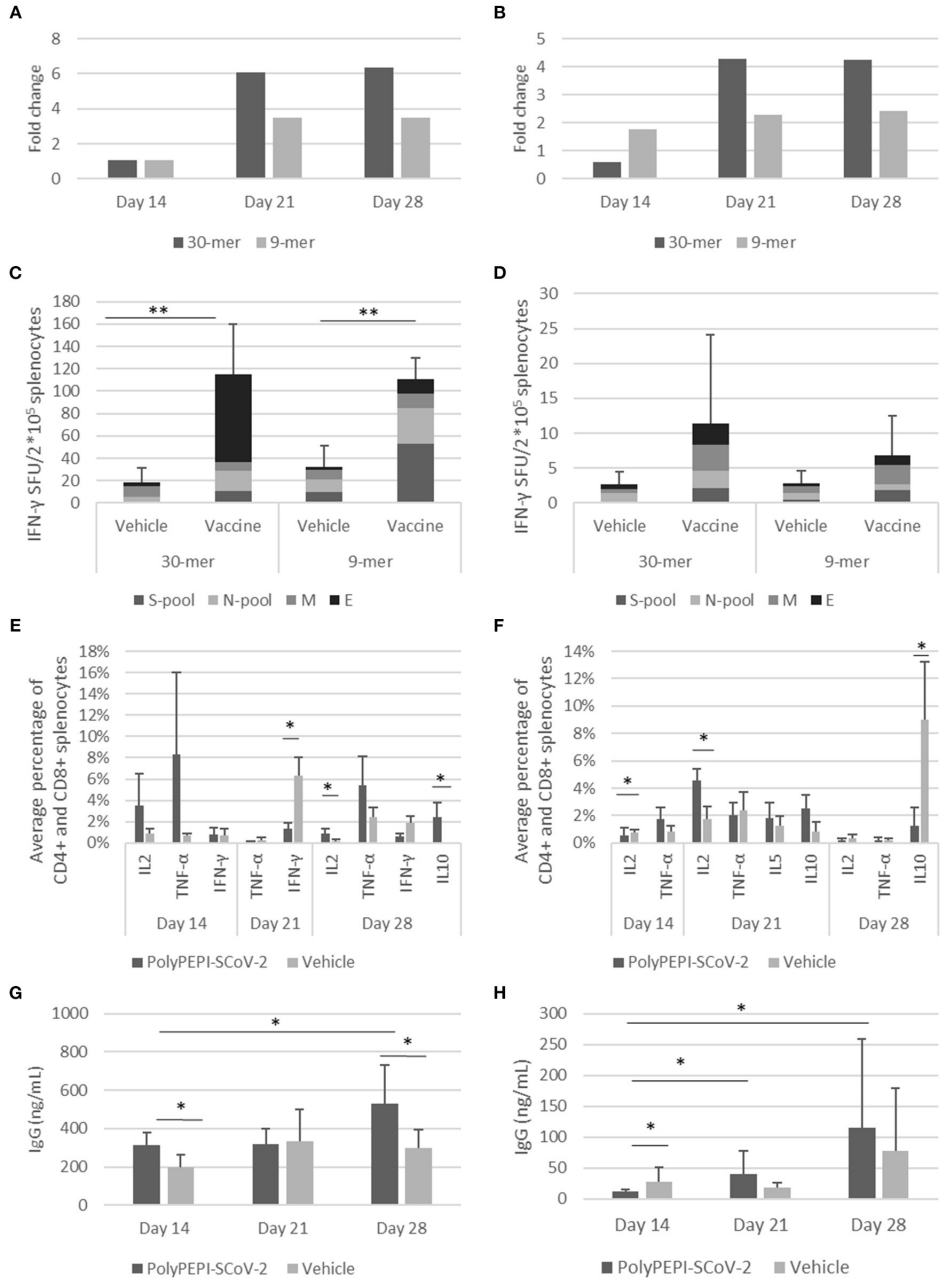
Induction of cellular and humoral immune responses by PolyPEPI-SCoV-2 vaccine in mouse models. Animals received PolyPEPI-SCoV-2 or Vehicle subcutaneously at days 0, 14. IFN-γ-producing T cell responses elicited by PolyPEPI-SCoV-2 expressed as fold change in BALB/c **(A)** and Hu-mouse **(B)** models compared to the respective Vehicle cohorts; diversity of vaccine-induced T cell responses after two doses at day 28 in BALB/c **(C)** and Hu-mouse **(D)** models by *ex vivo* ELISpot. Test conditions: stimulation with 30-mer S-pool (three S-peptides), N-pool (four N-peptides), M-peptide, E-peptide, or 9-mer pools (s-pool, n-pool, e1, m1 peptides). For Fold change calculation the average dSFU values of the 30-mer and 9-mer stimulation conditions are pooled. **(E,F)** PolyPEPI-SCoV-2 induced Th1 response and no significant Th2 cytokine induction shown as average of vaccine-specific CD4^+^ and CD8^+^ T cells producing IL-2, TNF-α, IFN-γ, IL-5 or IL-10 in BALB/c **(E)** and Hu-mouse **(F)**, using Intracellular cytokine staining. Mean +/-SEM are shown. 2 × 10^5^ cells were analyzed, gated for CD45^+^CD3^+^CD4^+^/CD8^+^. Average percentage was obtained by pooling the background-subtracted values of the 30-mer stimulation conditions for each cytokine for CD4^+^ and CD8^+^ splenocytes. IgG production measured from the plasma of BALB/c **(G)** and Hu-mice **(H)**. *N* = 6 animals at each time point, *p < 0.05, **p < 0.001 (Mann-Whitney U).

In BALB/c mice at day 14, PolyPEPI-SCoV-2 vaccination did not induce more IFN-γ production than the Vehicle (DMSO/Water emulsified with Montanide), this latter resulting in unusually high response probably due to Montanide mediated unspecific responses. Nevertheless, at days 21 and 28, the second dose of PolyPEPI-SCoV-2 increased IFN-γ production compared to Vehicle control group by 6-fold and 3.5-fold for splenocytes detected with the 30-mer and 9-mer peptides, respectively (Figure 6A).

In immunodeficient Hu-mice at day 14, PolyPEPI-SCoV-2 vaccination increased IFN-γ production by 2-fold with splenocytes specific for the 9-mer pool of peptides, but no increase was observed with 30-mer-stimulated splenocytes. At days 21 and 28, the second dose of PolyPEPI-SCoV-2 boosted IFN-γ production by 4- and 2-fold with splenocytes detected with the 30-mer and 9-mer pools of peptides, respectively (Figure 6B). Importantly, both 9-mer-detected CD8^+^ T cells and 30-mer-detected CD4^+^ and CD8^+^ T cell responses were directed against all four viral proteins targeted by the vaccine in both animal models (Figures 6C,D; Supplementary Figures 9A–F). Since the Hu-mouse model was developed by transplanting human CD34^+^ hematopoietic stem cells to generate human antigen-presenting cells and T- and B-lymphocytes into NOD/Shi-scid/IL-2Rγ null immunodeficient mice, this model provides a real human immune system model (Brehm et al., 2013). Therefore, the robust multi-antigenic CD4^+^ and CD8^+^ T cell responses obtained in this model indicate that the vaccination resulted in properly processed and HLA-presented epitopes and subsequent antigen-specific T cell responses by the human immune cells of the Hu-mice.

ICS assay was performed to investigate the polarization of the T cell responses elicited by the vaccination. Due to the low frequency of T cells, individual peptide-specific T cells were more difficult to visualize by ICS than by ELISpot, but a clear population of CD4^+^ and CD8^+^ T cells producing Th1-type cytokines of TNF-α and IL-2 were detectable compared to animals receiving Vehicle in both BALB/c and Hu-mouse models (Figures 6E,F; Supplementary Figures 10, 11). For IL-4 and IL-13 Th2-type cytokines, analysis did not reveal any specific response at any time point. Low levels of IL-5 and/or IL-10 cytokine-producing CD4^+^ T cells were detected for both models but it was significantly different from Vehicle control only for BALB/c mice at day 28. Even for this cohort the Th1/Th2 balance remained shifted toward Th1 for five out of six mice (one outlier) confirming an overall Th1-skewed T cell response elicited by the vaccine (Supplementary Figure 11).

PolyPEPI-SCoV-2 vaccination also induced humoral responses, as measured by total mouse IgG for BALB/c and human IgG for Hu-mouse models. In BALB/c mice, vaccination resulted in vaccine-induced IgG production after the first dose (day 14) compared with Vehicle control group. IgG elevation were observed for both BALB/c and Hu-mouse models at later time points after the second dose (Figures 6G,H). IgG levels measured from the plasma of Hu-mice (average 115 ng/mL, Figure 6H) were lower than for BALB/c (average 529 ng/mL, Figure 6G) at D28. This is consistent with the known limitation of the NOD/Shi-scid/IL-2Rγ null immunodeficient mouse regarding its difficulty generating the human humoral responses that lead to class-switching and IgG production (Brehm et al., 2013). Humanization rate of ~50% in the Hu-mouse model further reduces the theoretically expected IgG levels. Despite these limitations, the dose-dependent human IgG production indicates vaccine-generated human humoral responses. As expected, given that PolyPEPI-SCoV-2 peptides do not contain conformational B cell epitopes, vaccination did not result in measurable neutralizing antibodies as assessed from the sera of Hu-mice using PNA assay. A 50% Neutralizing Antibody Titer (NT50) was undetectable at the assay detection limit of 1:25 dilution, for each tested samples (data not shown).

## Discussion

We demonstrated that PolyPEPI-SCoV-2, a polypeptide vaccine candidate comprising nine synthetic long (30-mer) peptides derived from the four structural proteins of the SARS-CoV-2 (S, N, M, E) mimics the diversity of T cell immunity produced by natural SARS-CoV-2 infection, in each subject. The peptides were prospectively selected based on their frequency for an ethnically diverse, HLA-genotyped *in silico* cohort and their frequency was subsequently demonstrated in a group of convalescent subjects. Each (100%) selected peptide achieved an unprecedented recognition rate in 40–93% of convalescents, demonstrating their immunoprevalence in COVID-19. In comparison a comprehensive screening of 5,600 predicted epitopes restricted to 28 frequent HLA class I alleles in 99 COVID-19 convalescent subjects revealed 101/454 (22%) epitopes shared between at least two subjects (Tarke et al., 2021). As an external validation, T cells reactive to each of our peptides investigated (eight out of nine) were reported also for this larger cohort of convalescents, 62% of subjects having *ex vivo* recall responses specific to one or more PolyPEPI-SCoV-2 peptides (Tarke et al., 2021).

On the individual level, the PolyPEPI-SCoV-2-specific T cell repertoire used for recovery from asymptomatic/mild COVID-19 was extremely diverse: each donor had an average of seven different peptide-specific T cell pools, with multiple targets against SARS-CoV-2 proteins; 87% of donors had targets against at least three SARS-CoV-2 proteins and 53% against all four, 1–5 months after their disease onset. Despite 87% of subjects had CD8^+^ T cells against S protein, we found that S-specific (memory) T cells represented only 36% of the convalescents' total T cell repertoire detected with our peptides; the remaining 64% was distributed almost equally among N, M, and E proteins. These data support the increasing concern that S protein-based candidate vaccines are not harnessing the full potential of human anti-SARS-CoV-2 T cell immunity, especially since diversity of T cell responses was associated with mild/asymptomatic COVID-19 and they are vital for long-term immunity.

We demonstrated that individuals' anti-SARS-CoV-2 T cell responses reactive to the PolyPEPI-SCoV-2 peptide set are HLA genotype-dependent. Specifically, predicted, multiple autologous HLA binding epitopes (PEPIs) determine antigen-specific CD8^+^ T cell responses with 84% accuracy. This suggests, that PEPIs overcome the unexplained high false positive rates generally observed using only the epitope-binding affinity as the T cell response predictor (Lorincz et al., 2019; Toke et al., 2019; Nelde et al., 2020; Wells et al., 2020; Tarke et al., 2021). Particularly, this predictive value compares favorably to the 10–25% positive epitope-specific T cell tests obtained in HLA-matched COVID-19 subjects reported by two recent publications (Nelde et al., 2020; Tarke et al., 2021).

Our vaccine design concept, targeting multi-antigenic immune responses at both the individual and population level, represents a novel target identification strategy that has already been used successfully in cancer vaccine development to achieve unprecedented immune response rates correlating with initial efficacy in the clinical setting (Hubbard et al., 2019). For COVID-19, we focused on selecting fragments of the SARS-CoV-2 proteins that contain overlapping HLA class I and II T cell epitopes that can generate diverse and broad immune responses against the whole virus. Therefore, we selected long 30-mer fragments to favor generation of multi-antigenic effector responses (B cells and cytotoxic T cells) and helper T cell responses.

PolyPEPI-SCoV-2 vaccine elicits the desired humoral responses as well as the CD8^+^ and CD4^+^ T cells responses against all four SARS-CoV-2 proteins in vaccinated BALB/c and humanized mice. Particularly, the robust, truly vaccine-induced immune responses obtained in the humanized mice suggest that immune responses obtained in mice are relevant also in humans.

The interaction between T and B cells is a well-known mechanism toward both antibody-producing plasma cell production and generation of memory B cells (Parker, 1993). During the analysis of convalescents' antibody subsets, we found correlations between antigen-specific IgG levels and corresponding peptide-specific CD4^+^ T cell responses. This correlation might represent the link between CD4^+^ T cells and antibody production, a concept also supported by total IgG production in the animal models. Binding IgG antibodies can act in cooperation with the vaccine induced CD8^+^ killer T cells upon later SARS-CoV-2 exposure of the vaccinees. This interplay might result in effective CD8^+^ T cell mediated direct killing of infected cells and IgG-mediated killing of virus-infected cells and viral particles, inhibiting Th2-dependent immunopathologic processes, too.

In this way, it is expected that both intracellular and extracellular virus reservoirs are attacked to help viral clearance in the early stage of infection blocking progression to severe COVID-19, even in the absence of neutralizing antibodies (Parker, 1993; Kar et al., 2020).

This hypothesis may be supported by previous animal challenge studies demonstrating that reactivated T cells provided protection from lethal dose infection with SARS (Zhao et al., 2010; Channappanavar et al., 2014a). Moreover, a study reported that CD8^+^ T cells contribute to the protection of convalescent macaques against re-challenge with SARS-CoV-2 in the setting of waning and subprotective antibody titers (McMahan et al., 2020). For mRNA-based COVID-19 vaccines it was suggested that binding antibodies and T cell responses are responsible for early protection against COVID-19 and lack of neutralizing antibodies indicate they are not absolutely required for protection (Kalimuddin et al., 2021). As of yet, the role of T cell responses in the protection against SARS-CoV-2 infection or COVID-19 has not been directly demonstrated. We acknowledge that, historically, T cell-focused vaccines represent an uncharted territory in the development of highly effective vaccines where antibody-based vaccines already demonstrated major role. However, the pandemic is still evolving and it would be important to understand the body's response to infection and to vaccines in order to develop the most effective vaccine or vaccination strategy.

Although PEPIs generally underestimated the subject's overall T cell repertoire, they are precise target identification “tools” and predictors of PEPI-specific immune responses. In addition both predicted PEPI frequencies and related T cell response frequencies obtained for the convalescent cohort were in good alignment with the predicted PEPI frequencies obtained for the *in silico* model population used for vaccine design. Therefore, our findings could be extrapolated to large cohorts of 16,000 HLA-genotyped individuals and 16 human ethnicities, representative for global population. Based on this, PolyPEPI-SCoV-2 will likely generate meaningful, multi-antigenic CD8^+^ and CD4^+^ T cell responses in ~98% of the global population, independent of ethnicity. In comparison, a T cell epitope-based vaccine design approach based on globally frequent HLA alleles, as proposed by others, would miss generation of immune responses for ~50% of Black Caribbean, African, African-American, and Vietnamese ethnicities. We propose, HLA-genotypes should be taken into consideration during the development of widely desired, second-generation, “universal” vaccines focusing not only on humoral but cellular responses, too (Dai and Gao, 2021). We believe, focusing on several targets in each subject would better recapitulate the natural T cell immunity induced by the virus, potentially leading to long-term memory responses and protection against mutations.

The present study has limitations. The limited number of donors studied did not allow validation of the performance of our approach. Further statistically powered studies would be required to demonstrate immunoprevalence of the selected peptides in large cohorts of convalescents with different ethnic background, immune status, age, etc. Nevertheless, the study presents a novel *in silico* approach for the selection of immunogenic epitopes for an individual or a population, and promising initial confirmation at both individual and population level in two independent convalescent cohorts. Translation of predicted anti-SARS-CoV-2-specific T cell responses based on HLA- genotypes to T cell responses obtained upon vaccination should be carefully interpreted even in the light of immunogenicity data obtained in vaccinated animals modeling human immune system. HLA-genotype-dependent vaccine-specific T cell responses can be validated in a clinical study involving HLA-genotyped individuals.

Due to the well-known limitations of the NOD/Shi-scid/IL-2Rγ null immunodeficient mouse model for producing robust IgG antibodies, we opted to measure total IgG as demonstration of the vaccine's capacity to induce humoral responses in human immune systems. Therefore, split to individual SARS-CoV-2 antigen-specific antibody responses need to be confirmed in further (preferably human-like or human) models. Similarly, this study does not provide evidence for the pre-clinical efficacy of the vaccine. A challenge study will be performed in rodent model to investigate the impact of the vaccine-induced T and B cell responses on functional immunity and on the disease pathology upon SARS-CoV-2 exposure.

Synthetic polypeptide-based platform technology is considered a safe and immunogenic subunit vaccination strategy with several advantages over platforms using whole antigens: limits unwanted antigenicity, induces robust cellular responses and can be less reactogenic (Wu et al., 2008; Atsmon et al., 2012; Crooke et al., 2020; Kanduc and Shoenfeld, 2020; Poland, 2020; Vojdani and Kharrazian, 2020). Synthetic peptide manufacturing at multi-kilogram scale is relatively inexpensive and peptides are generally stable for years (>6 months stability demonstrated for PolyPEPI-SCoV-2). Therefore, the manufacturing and distribution of peptide vaccines could benefit from the well-established processes of the existing multinational or nation-sized facilities. Peptide-based vaccines have had only limited success to date, but this can be attributed to lack of knowledge regarding which peptides to use. Such uncertainty is reduced by an understanding of how an individual's genetic background is able to respond to specific peptides. As we demonstrated here, this knowledge drives to the desired and predicted immune responses, both on individual and population level.

In conclusion, our multi-antigen targeting peptide set has the potential to lead to a versatile second-generation tool against COVID-19. Potential clinical opportunities include: PolyPEPI-SCoV-2 used either alone or in combination with other vaccines focused on neutralizing-antibody responses in COVID-naïve subjects or used as a booster agent to broaden or strengthen immune responses in vaccinated or COVID-convalescent subjects, or used in early infection or in “long COVID” (therapeutic setting) or as a diagnostic tool in monitoring SARS-CoV-2-specific T cell responses. In addition, “*in silico* clinical trial” in large, ethnically diverse cohorts allows for continuous and rapid monitoring of the global coverage and cross-protection with the appearance of new viral variants, potentially de-risking the success of clinical trials and likely an indispensable tool for global post-vaccination surveillance.

## Data Availability Statement

The datasets presented in this study can be found in online repositories. The names of the repository/repositories and accession number(s) can be found in the article/Supplementary Material.

## Ethics Statement

Blood samples were collected from convalescent individuals (n = 15) at an independent medical research center in The Netherlands under an approved protocol (NL57912.075.16.) or collected by PepTC Vaccines Ltd (n = 2). All donors including the non-exposed individuals (n = 10) provided written informed consent to participate in this study. The study was conducted in accordance with the Declaration of Helsinki. The animal study was reviewed and approved by the French Ethical Committe (CEEAG) and validated by the French Ministry of Research.

## Author Contributions

ES designed and coordinated the preclinical experiments and participated in data evaluations. ZC designed the PolyPEPI-SCoV-2 vaccine and participated in preclinical data evaluations. LM and JT performed the *in silico* analyses and prepared the Figures and Tables of the manuscript. SP, JS, and AM performed the *in vitro* experiments using COVID-19 donors' specimen and participated in the analysis of these data. OL had leading role in the manufacturing and quality control of the PolyPEPI-SCoV-2 peptides and development of vaccine formulation. LM and IM performed the statistical analysis. KP and PP participated in data mining and literature search. ET participated in the design of the experiments and interpretation of data as well in the preparation of the manuscript. All authors reviewed the manuscript.

## Conflict of Interest

ES, ZC, LM, OL, JT, IM, KP, PP, and ET hold shares in Treos Bio Ltd. and are employed by Treos Bio Zrt. SP, JS and AM are employed by ImmunXperts SA, a Nexelis company. Authors at Treos Bio Ltd. are listed as inventors of the following patents: US10973909B1 and PCT/GB2021/050829.

## Footnotes

^1^Demographics of the world https://www.quora.com/What-are-all-the-races-and-their-world-population-demographics-the-entire-world.

^2^U.S. National Library of Medicine Severe acute respiratory syndrome coronavirus 2 https://www.ncbi.nlm.nih.gov/genome/browse#!/viruses/86693/.

^3^WHO. Retrieved 6 May 2021. “COVID-19 Weekly Epidemiological Update.”
