# Supplementary material for: A Peptide Vaccine Candidate Tailored to Individuals' Genetics Mimics the Multi-Targeted T Cell Immunity of COVID-19 Convalescent Subjects
Source: Front Genet. 2021 Jun 23;12:684152. doi: 10.3389/fgene.2021.684152 (PMC8261158; doi:10.3389/fgene.2021.684152)
Supplement: Supplementary file 1 [file Data_Sheet_1.docx]

Supplementary Material

## Supplementary Figures S1-S11

## Supplementary Tables S1-S7

##
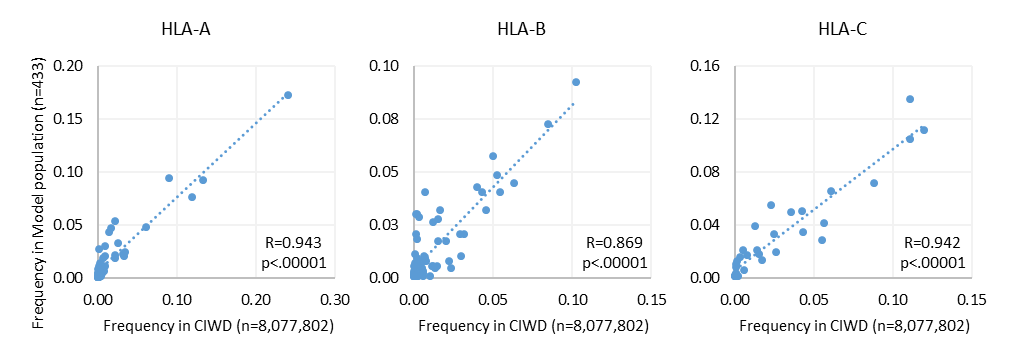


**Supplementary Figure S1. Allele frequency distributions in the Model Population representative for global allele frequencies.** HLA allele frequencies in the Model Population represent similar distribution as the allele frequencies of >8 million HLA-genotyped subjects in the CIWD database. CIWD 3.0 : Common (>=1 in 10,000), Intermediate (>=1 in 100,000) and Well Documented (>=5 occurrence), Rare (< 5 occurence) HLA alleles (database released in 2020). R-Pearson correlation coefficient. Related to Supplementary Table S3.

A

**
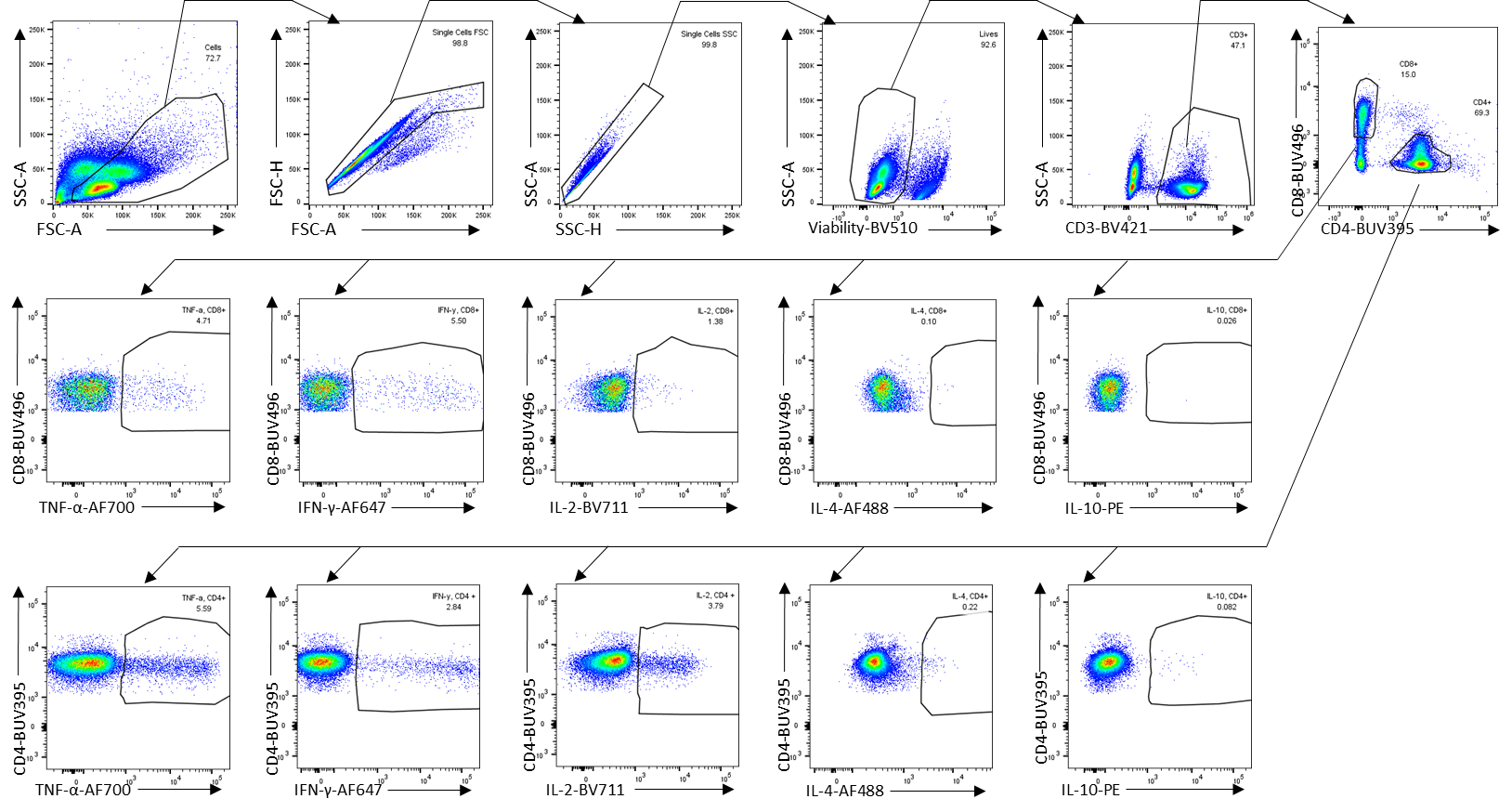
**

| B |
| --- |
| 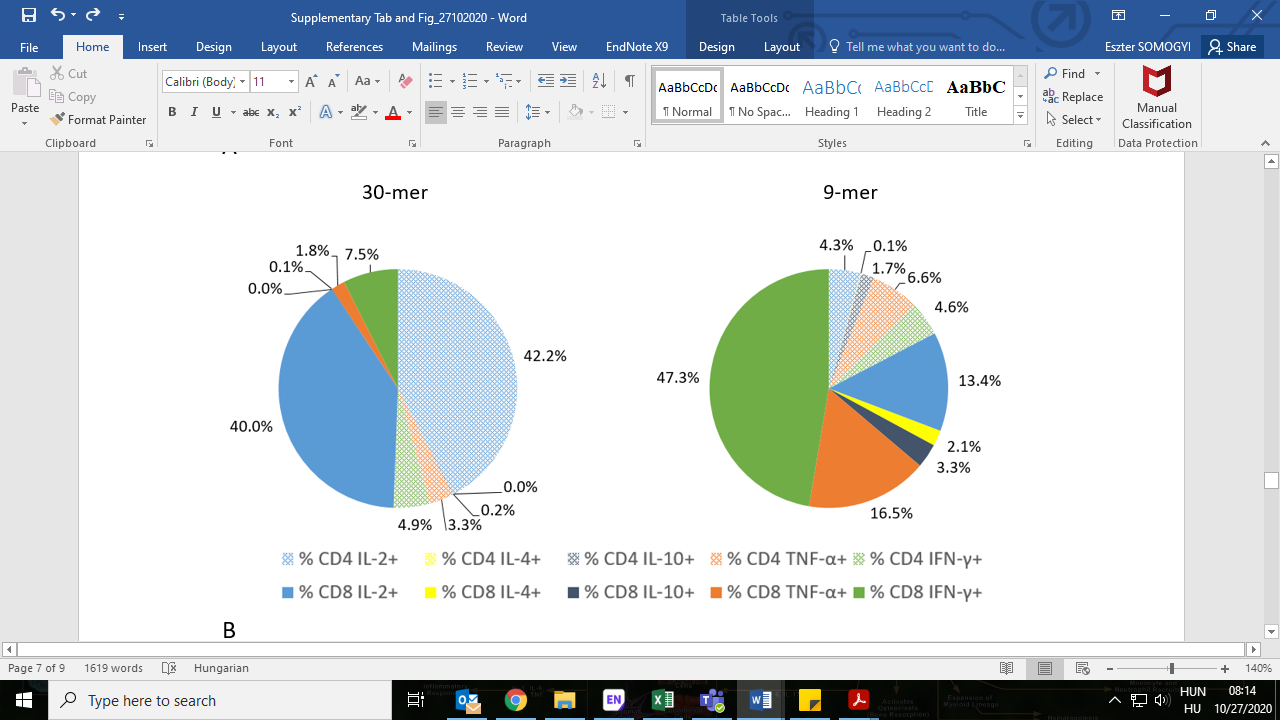 |
| c |
|  |

**Supplementary Figure S2. Cytokine production by COVID-19 convalescents’ T cells reactive to PolyPEPI-SCoV-2 peptides determined *ex vivo* from their PBMC by intracellular staining assay.** (**A**) Gating strategy applied for PBMC in the Intracellular Cytokine Staining analysis. (**B**) Cytokine profile of CD4^+^ and CD8^+^ T cells+ obtained by stimulations with 9-mer and 30-mer peptides (n=17). (**C**) Th1 dominance in vaccine-specific T cells stimulated with 30-mer peptides.

**Supplementary Figure S3. IFN-γ^+^ T cell responses detected for COVID-19 convalescent donors against the 9-mer peptides (PEPI hotspots) of PolyPEPI-SCoV-2 vaccine measured by enriched FluoroSpot assay.** s2, s5, and s9 are the three S-specific 9-mer peptide sequences derived from the Spike-specific vaccine 30-mers. n1–n4 are the four Nucleoprotein-specific 9-mer peptide sequences derived from the N-specific vaccine 30-mers. e1 and m1 are Envelope and Membrane-specific 9-mer peptide sequences derived from the E or M-specific vaccine 30-mers, respectively (Table 1 Bold). dSFU, delta spot forming units calculated as non-stimulated background corrected spot counts per 10^6^ PBMC. Average and individual data for each subject are presented, n=15. PBMC, peripheral blood mononuclear cells.


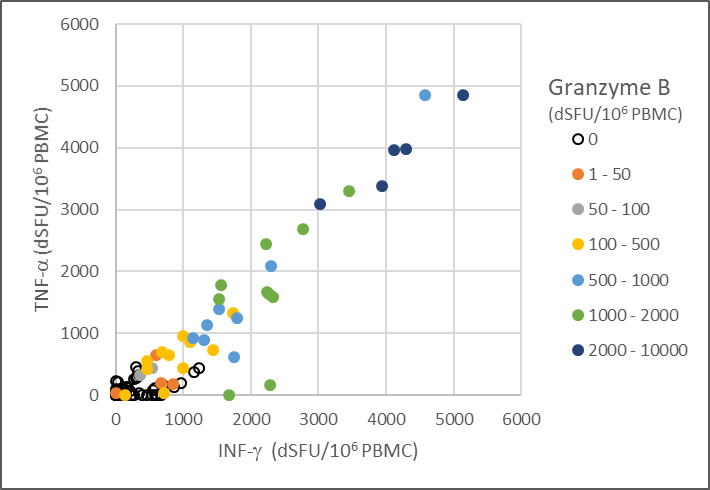


**Supplementary Figure S4. PolyPEPI-SCoV-2-specific polyfunctional T cells detected in COVID-19 convalescents’ blood.** IFN-γ and/or TNF-α and/or Granzyme-B positive T cell responses detected for each patient with individual 9-mer peptide stimulations using enriched FluoroSpot assay. dSFU stands for delta spot forming units, calculated as non-stimulated background corrected spot counts per 10^6^ PBMC; n=15.

**
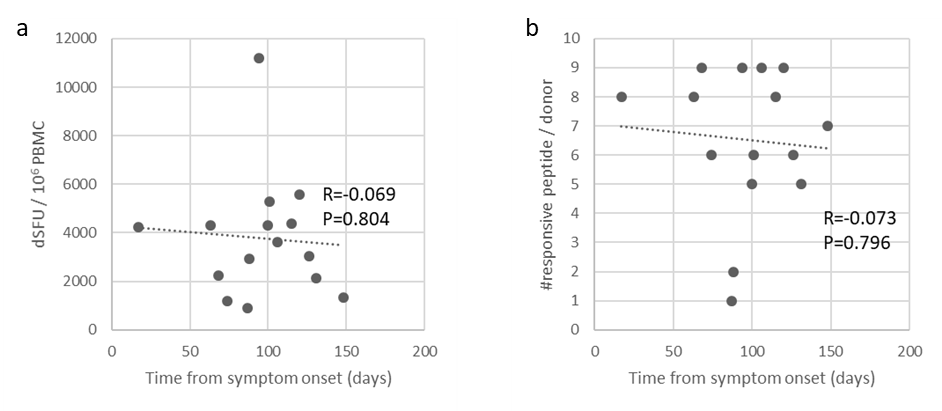
**

B

A

**Supplementary Figure S5. Magnitude and breadth of COVID-19 convalescent donors’ T cell responses relative to time from symptom onset.** (**A**) Magnitude of PolyPEPI-SCoV-2-reactive T cell responses (**B**) Breadth of vaccine peptide-reactive CD8^+^ T cell responses of convalescent donors, detected with enriched FluoroSpot assay with individual 9-mer stimulations. dSFU stands for delta spot forming units, calculated as background corrected spot counts per 10^6^ PBMC. n=15; R-Pearson correlation coefficient.


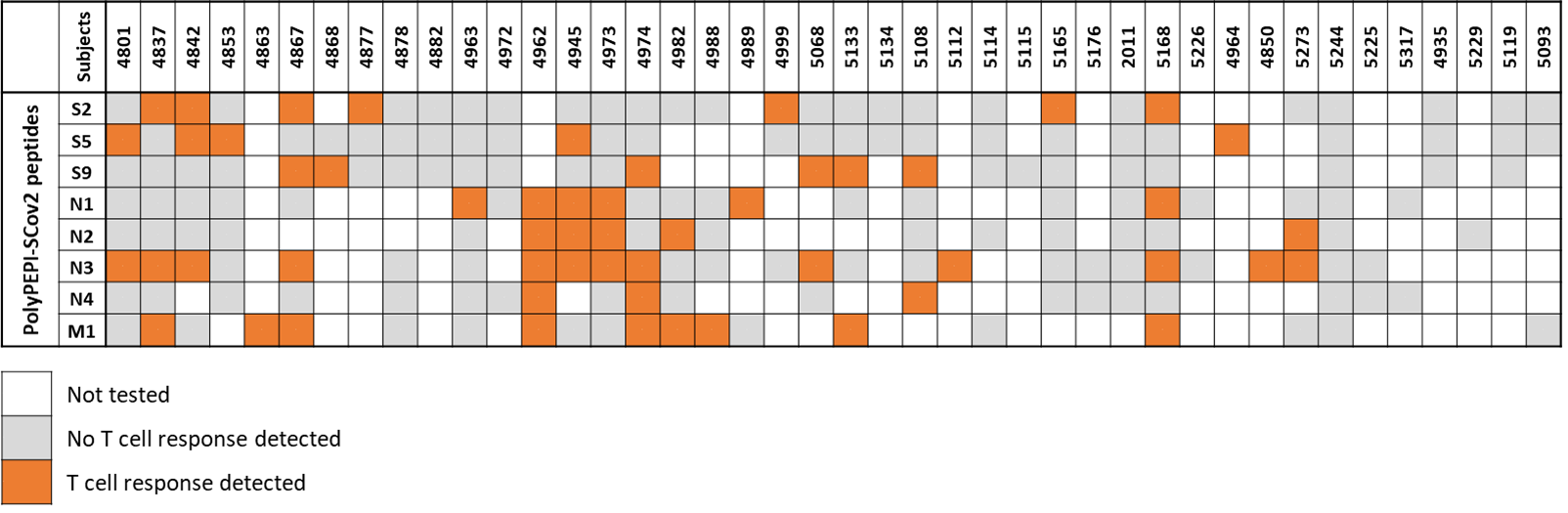


**Supplementary Figure S6.** Analysis of convalescents’ T cell reactivities measured with epitopes overlapping with PolyPEPI-SCoV-2 peptides, as reported by Tarke et al (Tarke et al. 2021). 159 MHC I or MHC II epitopes (8-20mers) tested for 42 COVID-19 convalescents had overlapping sequences (at least 8 consecutive amino acids) with eight of nine PolyPEPI-SCoV-2 peptides.


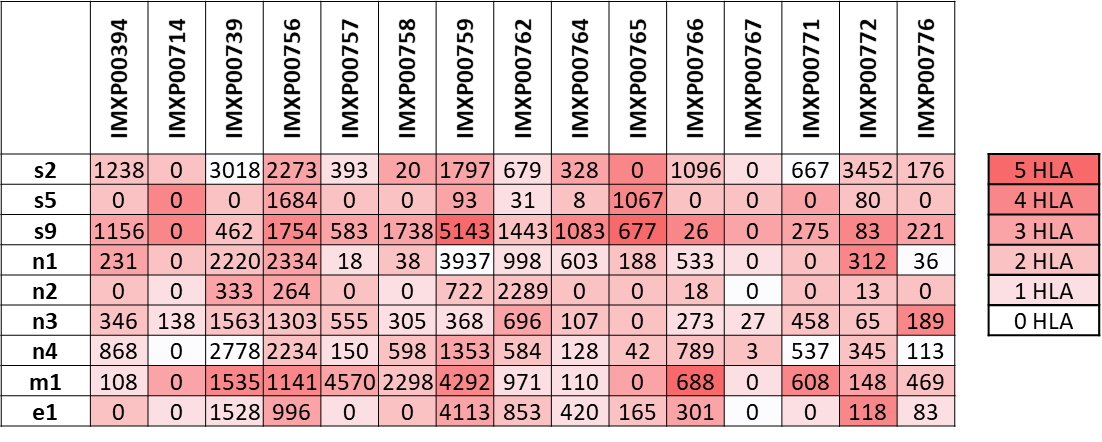


**Supplementary Figure S7.** Matching predicted multiple autologous HLA binding epitopes (n=9) with the same peptide-reactive CD8+ T cell responses in n=15 donors (135 data points). Numbers denote dSFU determined by enriched FluoroSpot assay. Colour codes refer to the predicted number of autologous HLA alleles binding the specific peptides. dSFU, delta spot forming units calculated as non-stimulated background corrected spot counts per 10^6^ PBMC.

**
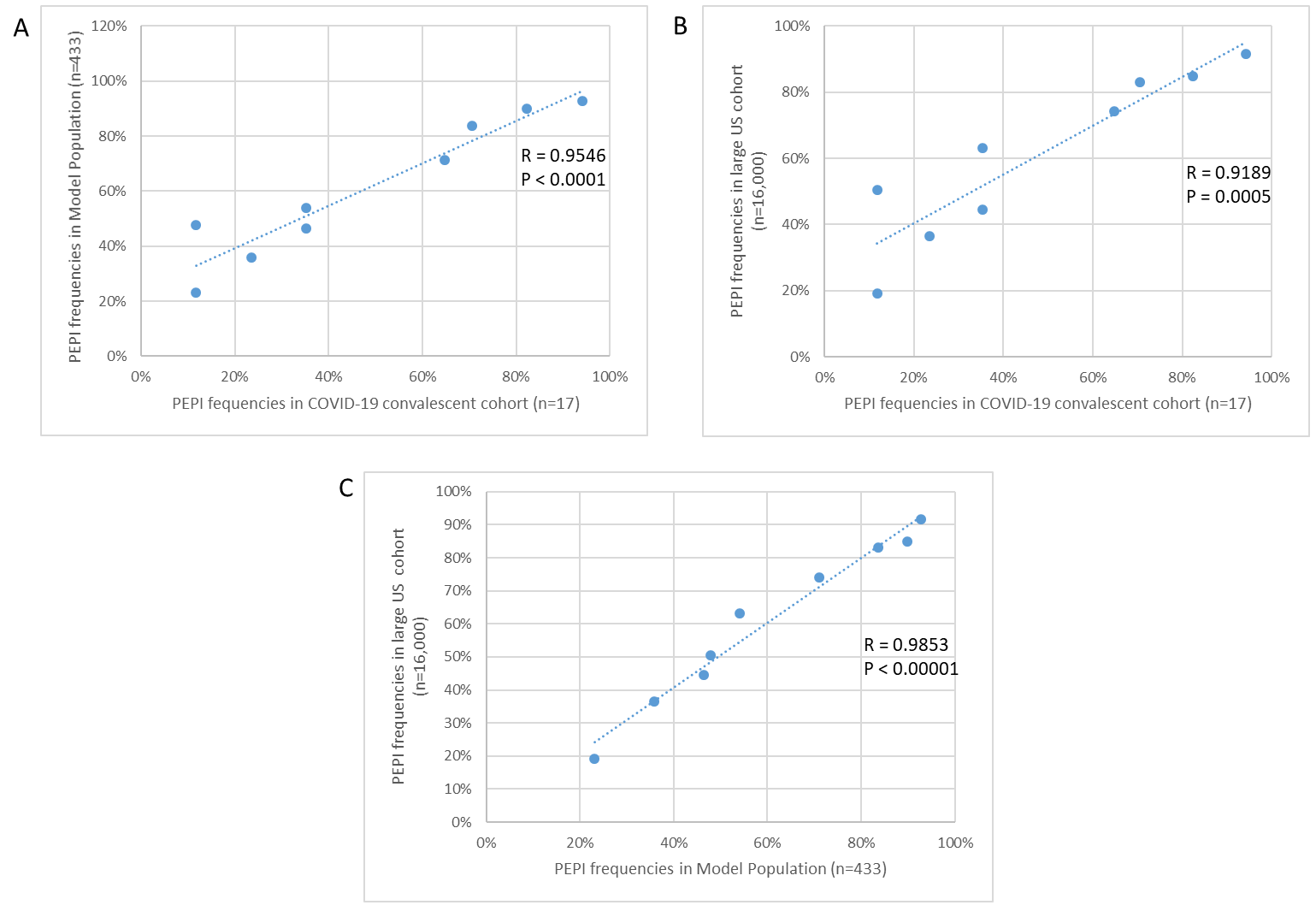
**

**Supplementary Figure S8. Comparison of** predicted PolyPEPI-SCoV-2 peptide-specific PEPI frequencies of different populations with complete HLA class I genotype. PEPI frequencies of the COVID-19 convalescent cohort (n=17) against the Model Population (n=433) (**A**) and against the large US cohort (n=16,000) (**B**) and of the Model Population compared to the large US cohort (n=16,000) (**C**). R – Pearson correlation coefficient.


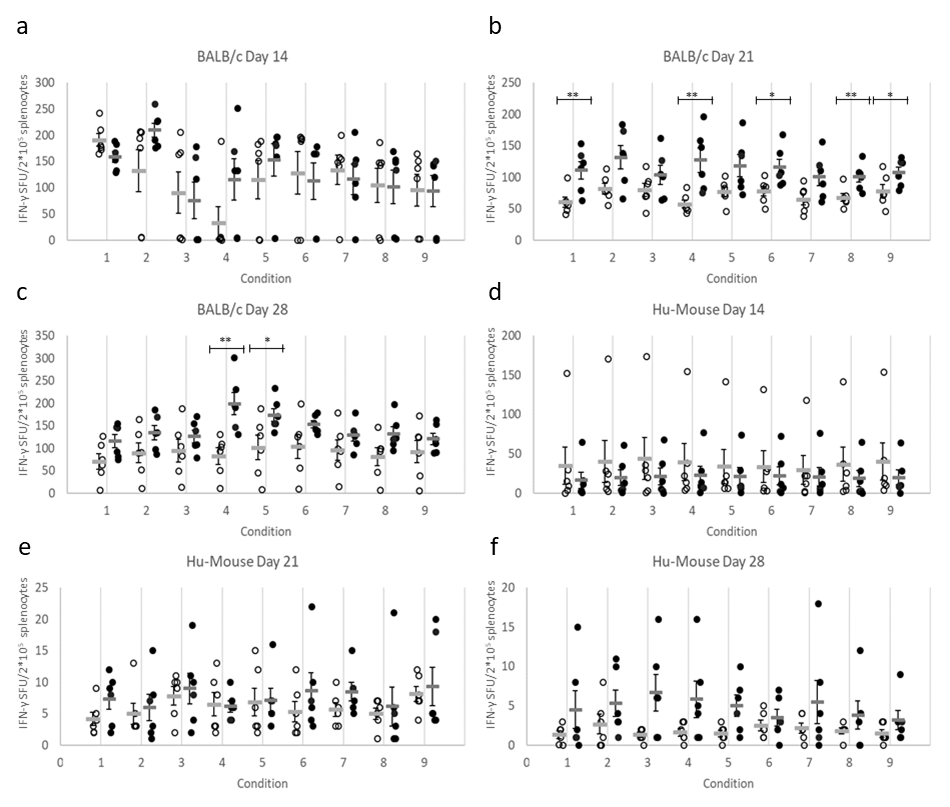


F

E

D

C

B

A

**Supplementary Figure S9. The PolyPEPI-SCoV-2 treatment increases IFN-γ-producing T cells in mice.** PolyPEPI-SCoV-2 vaccinated mice (black dots) compared to Vehicle (DMSO/Water-Montanide) control animals (white circles). IFN-γ production was analyzed by *ex vivo* ELISpot in the spleen after re-stimulation with peptides at day 14 (**A** BALB/c; and **D**, Hu-mice), day 21 (**B**, BALB/c; and **E**, Hu-mice), and day 28 (**C**, BALB/c; and **F**, Hu-mice). Conditions: 1: S-pool; Spike-specific 30-mer pool of S2, S5, and S9 peptides. 2: N-pool; Nucleoprotein-specific 30-mer pool of N1, N2, N3, and N4 peptides. 3: M1 Membrane-specific 30-mer peptide. 4: E1 Envelope-specific 30-mer peptide. 5: S-pool; Spike-specific 9-mer pool of s2, s5, s9 HLA class I PEPI hotspot fragment of the corresponding 30-mers. 6: N-pool; Nucleoprotein-specific 9-mer pool of n1, n2, n3, and n4 HLA class I PEPI hotspot fragment of the corresponding 30-mers. 7: m1 Membrane-specific 9-mer HLA class I PEPI hotspot fragment of the corresponding 30-mer. 8: e1 Envelope-specific 9-mer HLA class I PEPI hotspot fragment of the corresponding 30-mer. 9: unstimulated control. Individual spot forming units (SFU) and means are shown and represent spots per 2×10^5^ splenocytes. n=6 mice per group were analyzed. Statistical analysis was performed by Mann-Whitney test. *, p<0.05; **, p<0.01.


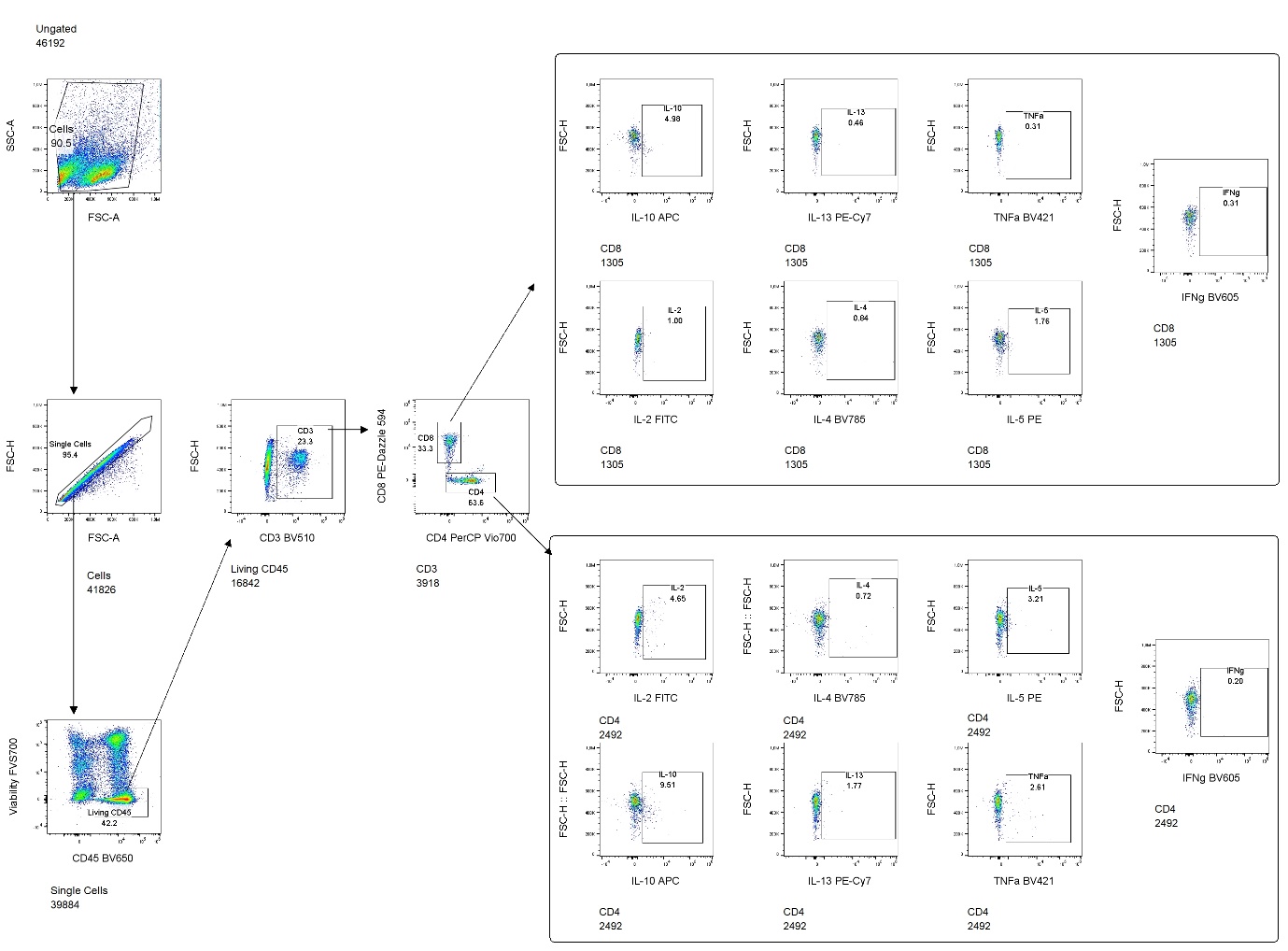


**Supplementary Figure S10. Gating strategy applied for splenocytes in the Intracellular Cytokine Staining analysis by flow cytometry during BALB/c mice and Hu-mice study.**


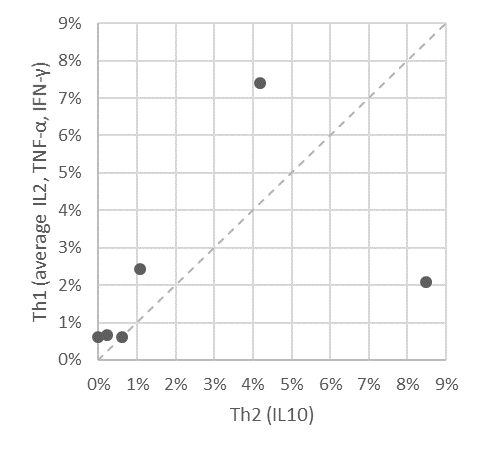


**Supplementary Figure S11. Th1/Th2 balance for T cells detected with PolyPEPI-SCoV-2 vaccine in BALB/c mice at day 28.** Average CD4^+^ and CD8^+^ T cells producing IL2, TNF-α, IFN-γ (Th1 cytokines) and IL10 (Th2 cytokine) for each immunized mice (n=6) using ICS assay. 2×10^5^ cells were analyzed, gated for CD45^+^ cells, CD3^+^ T cells, CD4^+^ or CD8^+^ T cells. The average percent was obtained by pooling the background subtracted values of the 4 stimulation conditions (30-mer S-pool, N-pool, E1 and M1 peptides) for each cytokine for CD4^+^ and CD8^+^ T cells.

**Supplementary Table S1. COVID-19 convalescent donor information.** All donors were caucasoid, with mild/asymptomatic disease and no hospitalization (except one, marked with *). S/Co, sample/control ratio; values were determined according to the manufacturer’s instructions, and test results are interpreted as negative in S/Co <0.9, not conclusive if S/CO = 0.9–1.1, and positive if S/Co >1.1. COI, cut-off index; values were determined according to the manufacturer’s instructions, and test results are interpreted as negative in COI <0.9, inconclusive with COI 0.9–1.1, and positive if COI >1.1. NA, data not available. Italic, negative or inconclusive values. ** Complaints: a, cough; b, sore throat; c, fever; d, short of breath; e, stomach/intestinal complaints; f, chest pain; g, sore eyes; h, odor or taste loss; i, headache; j, fatigue; k, other complaints (pulmonary embolism and cardiac arrest for IMXP00759; leg pain, arm pain, muscle pain, pain in the eyes).

| Donor ID | Gender | Complaints from/ to:  (as reported by the donors) | Complaints** | Blood collection date | Time from first symptom to blood collection | IgA | IgM | IgG | IgG-S1 | IgG-N |
| --- | --- | --- | --- | --- | --- | --- | --- | --- | --- | --- |
|  |  |  |  |  |  | DiaPro ELISA | | | EUROIMMUN | ROCHE |
|  |  |  |  |  |  | S/Co | S/Co | S/Co | S/Co | COI |
| IMXP00394 | Female | 30 March 2020 - 20 April 2020 | a,b,c,d,h,i,j | 4-Aug-20 | 126 days | *0.36* | 5.065 | 5.752 | 4,48 | 54.38 |
| IMXP00714 | Male | 1 May 2020 – 15 May 2020 | a,b,c,h,i,j,k | 27-Jul-20 | 87 days | 1.324 | 8.524 | 11.524 | 5,35 | 73.06 |
| IMXP00739 | Female | 30 April 2020 | j | 2-Jun-20 | 63 days | *0.929* | 8.841 | 11.967 | 4,54 | 77.61 |
| IMXP00756 | Female | 2 April 2020 - 12 April 2020 | b,c,d,f,i,j | 9-Jun-20 | 68 days | *0.989* | 4.606 | 12.193 | 3,56 | 78.47 |
| IMXP00757 | Female | 29February 2020 - 14 April 2020 | a,b,c,d,e,h,i,j | 9-Jun-20 | 101 days | 1.154 | 5.847 | 8.701 | 7,62 | 29.47 |
| IMXP00758 | Female | 2 April 2020 - 30 arpril 2020 | c,d,h,i,j | 15-Jun-20 | 74 days | 1.356 | 7.757 | 11.774 | 5,79 | 121.9 |
| IMXP00759* | Male | 13 March 2020 - 28 March 2020 | a,c,d,f,h,i,j,k | 15-Jun-20 | 94 days | 6.307 | 10.666 | 13.838 | 9,27 | 87.09 |
| IMXP00762 | Female | 15 March 2020 – 19 March 2020 | b,c,j | 29-Jun-20 | 106 days | 1.251 | 7.314 | 4.46 | 7,25 | 131.5 |
| IMXP00764 | Female | 16 March 2020 – 2 April 2020 | a,b,e,h,i,j,k | 6-Jul-20 | 115 days | 5.161 | 9.739 | 11.677 | 1,32 | 46.59 |
| IMXP00765 | Female | 29 March 2020 - 15 May 2020 | a,d,e,h,i,j,k | 7-Jul-20 | 100 days | *0.565* | 2.948 | 1.54 | 1,32 | 13.4 |
| IMXP00766 | Female | 20 June 2020 - 23 June 2020 | b,c,h,j | 7-Jul-20 | 17 days | *0.771* | 4.648 | 3.973 | 4,14 | 6.25 |
| IMXP00767 | Female | 10 April 2020 - 10 May 2020 | d,e,f,j,k | 7-Jul-20 | 88 days | *0.88* | 5.402 | 3.459 | 2,37 | 52.29 |
| IMXP00771 | Female | 18 March 2020 – 1 April 2020 | a,d,i,j | 28-Jul-20 | 131 days | *0.791* | 7.775 | 8.322 | 4,04 | 119.4 |
| IMXP00772 | Female | 30 March 2020 - 30 April 2020 | g,k | 28-Jul-20 | 120 days | 1.105 | 4.256 | 2.54 | 1,26 | 10.87 |
| IMXP00776 | Female | 9 March 2020 - 14 March 2020 | c,e,i,j,k | 4-Aug-20 | 148 days | *1.012* | 9.196 | 10.887 | 2,26 | 88.64 |
| PTC1 | Male | 15 April 2020 | e | 13-Jul-20 | 89 days | *0.53* | *0.41* | 2.63 | NA | 18.96 |
| PTC2 | Female | 15 April 2020 | e | 13-Jul-20 | 89 days | *0.45* | *0.35* | 1.49 | NA | 26.09 |

**Supplementary Table S2. Complete HLA genotype of convalescent donors.**

| Nr. | Donor ID | HLA-A | | HLA-B | | HLA-C | | DRB1 | | DQB1 | | DPB1 | |
| --- | --- | --- | --- | --- | --- | --- | --- | --- | --- | --- | --- | --- | --- |
| 1 | IMXP00394 | A*11:01 | A*24:02 | B*35:03 | B*55:01 | C*03:03 | C*12:03 | DRB1*01:01 | DRB1*13:01 | DQB1*05:01 | DQB1*06:03 | DPB1*04:01 | DPB1*04:02 |
| 2 | IMXP00714 | A*01:01 | A*02:01 | B*07:02 | B*44:03 | C*04:01 | C*07:02 | DRB1*07:01 | DRB1*15:01 | DQB1*02:02 | DQB1*06:02 | DPB1*01:01 | DPB1*04:01 |
| 3 | IMXP00739 | A*03:01 | A*03:01 | B*07:02 | B*35:03 | C*04:01 | C*07:02 | DRB1*14:54 | DRB1*15:01 | DQB1*05:03 | DQB1*06:02 | DPB1*02:01 | DPB1*10:01 |
| 4 | IMXP00756 | A*02:01 | A*11:01 | B*15:01 | B*55:01 | C*03:03 | C*03:04 | DRB1*14:54 | DRB1*15:02 | DQB1*05:03 | DQB1*06:01 | DPB1*04:01 | DPB1*04:01 |
| 5 | IMXP00757 | A*02:01 | A*31:01 | B*40:01 | B*44:02 | C*03:04 | C*05:01 | DRB1*04:01 | DRB1*15:01 | DQB1*03:01 | DQB1*06:02 | DPB1*04:01 | DPB1*04:01 |
| 6 | IMXP00758 | A*01:01 | A*11:01 | B*08:01 | B*44:02 | C*05:01 | C*07:01 | DRB1*03:01 | DRB1*12:01 | DQB1*02:01 | DQB1*03:01 | DPB1*01:01 | DPB1*02:01 |
| 7 | IMXP00759 | A*24:02 | A*30:01 | B*13:02 | B*57:01 | C*06:02 | C*06:02 | DRB1*07:01 | DRB1*07:01 | DQB1*02:02 | DQB1*03:03 | DPB1*04:01 | DPB1*17:01 |
| 8 | IMXP00762 | A*02:05 | A*30:02 | B*15:03 | B*51:01 | C*12:03 | C*14:02 | DRB1*07:01 | DRB1*07:01 | DQB1*02:02 | DQB1*02:02 | DPB1*04:01 | DPB1*04:01 |
| 9 | IMXP00764 | A*01:01 | A*23:01 | B*44:03 | B*49:01 | C*04:01 | C*07:01 | DRB1*07:01 | DRB1*08:01 | DQB1*02:02 | DQB1*04:02 | DPB1*03:01 | DPB1*04:01 |
| 10 | IMXP00765 | A*02:01 | A*29:02 | B*40:01 | B*44:03 | C*03:04 | C*16:01 | DRB1*07:01 | DRB1*08:01 | DQB1*02:02 | DQB1*04:02 | DPB1*03:01 | DPB1*11:01 |
| 11 | IMXP00766 | A*03:01 | A*30:01 | B*13:02 | B*27:05 | C*02:02 | C*06:02 | DRB1*07:01 | DRB1*14:01 | DQB1*02:02 | DQB1*05:03 | DPB1*04:01 | DPB1*04:01 |
| 12 | IMXP00767 | A*01:01 | A*03:02 | B*38:01 | B*51:01 | C*12:03 | C*15:02 | DRB1*04:02 | DRB1*13:01 | DQB1*03:02 | DQB1*06:03 | DPB1*02:01 | DPB1*09:01 |
| 13 | IMXP00771 | A*02:01 | A*03:01 | B*07:02 | B*35:03 | C*04:01 | C*07:02 | DRB1*08:01 | DRB1*15:01 | DQB1*04:02 | DQB1*06:02 | DPB1*04:01 | DPB1*04:02 |
| 14 | IMXP00772 | A*02:01 | A*26:01 | B*15:01 | B*55:01 | C*03:03 | C*03:03 | DRB1*13:01 | DRB1*13:01 | DQB1*06:03 | DQB1*06:03 | DPB1*03:01 | DPB1*03:01 |
| 15 | IMXP00776 | A*24:02 | A*68:01 | B*27:05 | B*35:01 | C*04:01 | C*07:02 | DRB1*04:01 | DRB1*15:01 | DQB1*03:01 | DQB1*06:02 | DPB1*02:01 | DPB1*04:02 |
| 16 | PTC1 | A*02:01 | A*24:02 | B*35:03 | B*51:01 | C*01:02 | C*04:01 | DRB1*01:01 | DRB1*08:01 | DQB1*03:02 | DQB1*05:01 | DPB1*04:01 | DPB1*04:02 |
| 17 | PTC2 | A*26:01 | A*32:01 | B*37:01 | B*40:02 | C*02:02 | C*06:02 | DRB1*11:04 | DRB1*16:02 | DQB1*03:01 | DQB1*05:02 | DPB1*04:01 | DPB1*10:01 |

**Supplementary Table S3. HLA coverage of alleles represented in Model Population.** African/African American (AFA), Asian/Pacific Islands (API), European/European descent (EURO), Middle East/North coast of Africa (MENA), South or Central America/Hispanic/Latino (HIS), Native American populations (NAM), Unknown/Not asked/Multiple ancestries/Other (UNK). CIWD 3.0 : Common (>=1 in 10,000), Intermediate (>=1 in 100,000) and Well Documented (>=5 occurrence), Rare (< 5 occurence) HLA alleles (database released in 2020). Related to Supplementary Figure S1.

| **HLA Class** | **Description** | **AFA**  N= 195,223 | **API**  N= 650,553 | **EURO**  N= 5,983,418 | **MENA**  N= 202,042 | **HIS**  N= 351,200 | **NAM**  N= 33,607 | **UNK**  N= 661,759 | **Total**  N= 8,077,802 |
| --- | --- | --- | --- | --- | --- | --- | --- | --- | --- |
| **HLA-A** | **Allele Count by Population Group in CIWD** | 388,476 | 1,291,125 | 11,929,417 | 402,447 | 700,632 | 66,971 | 1,320,493 | 16,099,561 |
| **Covered by Model population's HLA set (n=49)** | | 356,264 | 1,210,978 | 11,192,793 | 380,568 | 621,447 | 60,270 | 1,204,657 | 15,026,977 |
|  | **Coverage** | **98.2%** | **95.9%** | **99.3%** | **97.6%** | **97.0%** | **96.7%** | **98.0%** | **98.7%** |
| **HLA-B** | **Allele Count by Population Group in CIWD** | 388,579 | 1,298,351 | 11,941,489 | 402,160 | 700,912 | 66,967 | 1,320,714 | 16,119,172 |
| **Covered by Model population's HLA set (n=71)** | | 356,687 | 1,169,460 | 10,821,481 | 358,189 | 580,452 | 57,823 | 1,176,597 | 14,520,689 |
|  | **Coverage** | **96.4%** | **91.8%** | **96.1%** | **91.5%** | **88.6%** | **91.3%** | **94.2%** | **95.1%** |
| **HLA-C** | **Allele Count by Population Group in CIWD** | 389,619 | 1,255,403 | 11,827,887 | 403,229 | 690,043 | 67,072 | 1,302,662 | 15,935,915 |
| **Covered by Model population's HLA set (n=32)** | | 343,565 | 1,132,914 | 10,400,481 | 364,466 | 583,484 | 55,031 | 1,132,848 | 14,012,789 |
|  | **Coverage** | **98.9%** | **94.8%** | **99.2%** | **96.0%** | **96.2%** | **96.2%** | **98.5%** | **98.5%** |
| **HLA-A-B-C coverage by Model population’s HLA set (n=152):** | | | | | | | | | **97.4%** |
|  | | | | | | | | | |

**Supplementary Table S4. HLA alleles and frequencies in the large US cohort with 16 ethnicities (n=16,000)**

See xls Table in a separate file.

**Supplementary Table S5. Sequence alignment results between PolyPEPI-SCoV-2 and coronavirus strains.** Sequence comparison was made with 8-mer long peptide matching between the aligned protein sequence pairs, defined as the minimum length requirement for a CD8^+^ T cell epitope. Max. amino acid matching: the longest identical amino acid sequence length. Highlighted grey values represent identical sequences of at least eight amino acids.

| **Target Protein** | **PolyPEPI-SCoV-2 vaccine peptide sequences** | | **Common 8-mer % / Max. amino acid matching** | | | | | |
| --- | --- | --- | --- | --- | --- | --- | --- | --- |
|  |  |  | **229E** | **NL63** | **OC43** | **HKU1** | **MERS** | **SARS** |
| Spike | S2 | GVYYPDKVFRSSVLHSTQDLFLPFFSNVTW | 0% / 4 | 0% / 4 | 0% / 4 | 0% / 3 | 0% / 3 | 4% / 8 |
|  | S5 | DSSSGWTAGAAAYYVGYLQPRTFLLKYNEN | 0% / 3 | 0% / 4 | 0% / 4 | 0% / 4 | 0% / 5 | 0% / 4 |
|  | S9 | ALQIPFAMQMAYRFNGIGVTQNVLYENQKL | 0% / 4 | 0% / 4 | 0% / 4 | 0% / 4 | 0% / 5 | 96% / 29 |
| Nucleoprotein | N1 | RSKQRRPQGLPNNTASWFTALTQHGKEDLK | 0% / 3 | 0% / 3 | 0% / 3 | 0% / 3 | 0% / 6 | 78% / 25 |
|  | N2 | SKKPRQKRTATKAYNVTQAFGRRGPEQTQG | 0% / 4 | 0% / 4 | 0% / 6 | 0% / 7 | 0% / 4 | 65% / 17 |
|  | N3 | ELIRQGTDYKHWPQIAQFAPSASAFFGMSR | 0% / 3 | 0% / 4 | 0% / 3 | 0% / 4 | 0% / 5 | 96% / 29 |
|  | N4 | QRQKKQQTVTLLPAADLDDFSKQLQQSMSS | 0% / 5 | 0% / 3 | 0% / 4 | 0% / 3 | 0% / 3 | 9% / 9 |
| Membrane | M1 | LSYFIASFRLFARTRSMWSFNPETNILLNV | 0% / 5 | 0% / 6 | 4% / 8 | 4% / 8 | 4% / 8 | 78% / 25 |
| Envelope | E1 | NIVNVSLVKPSFYVYSRVKNLNSSRVPDLL | 0% / 4 | 0% / 4 | 0% / 3 | 0% / 5 | 0% / 4 | 35% / 12 |

**Supplementary Table S6. Response rate of COVID-19 convalescent donors to one, two, three, or all four viral antigens targeted by the PolyPEPI-SCoV-2 vaccine, as measured by *ex vivo* FluoroSpot assay.** Nine-mers are the hotspot HLA class I PEPIs embedded within each 30-mer vaccine peptide coresponding to the four structural proteins: S, Spike; N, Nucleoprotein; M, membrane; E, envelope proteins.

| **Number of reactive antigens (S, N, M, E)** | **Percentage of subjects responsive to 30-mer peptides (N=17)** | **Percentage of subjects responsive to 9-mer peptides (N=17)** |
| --- | --- | --- |
| 1 | 94% | 100% |
| 2 | 82% | 53% |
| 3 | 59% | 18% |
| 4 | 18% | 6% |

Supplementary Table S7A Safety analysis, clinical score data table of BALB/c mice. Clinical safety scores were established by characterization of five different clnical signs (coat, movement, activity, paleness, body weight), according to the following specification: Coat: score 0 – normal; score 1– lack of grooming, partial alopecia; score 2 – massive alopecia, wounds, bleedings, inflammation. Movement: score 0 – normal; score 2 – slow movement, paralysis of one animal; score 3 – difficulties to eat and drink, paralysis to more than one animal. Activity: score 0 – normal; score 1 – agitated, over-reactive, hypo-reactive; score 3 – prostrated. Paleness: score 0 – normal; score 1 – slight (no ear vessels visible); score 2 – severe (ears plus feet affected). Body weight: score 0 – normal; score 2 – segmentation of the vertebral column evident, pelvic bones palpable; score 3 – sceletal strucure prominent. Maximum cumulative clinical score allowed: 6. n.a.: not applicable.

| **Days after 1^st^ vaccination:** | | | **-2** | **6** | **12** | **19** | **26** |
| --- | --- | --- | --- | --- | --- | --- | --- |
| **Mouse strain** | **Mouse ID** | **treatment** | **Cumulative clinical score** | | | | |
| BALB/c | 1 | PolyPEPI-SCoV-2 | 0 | 0 | 0 | n.a. | n.a. |
|  | 2 |  | 0 | 0 | 0 | n.a. | n.a. |
|  | 3 |  | 0 | 0 | 0 | n.a. | n.a. |
|  | 4 |  | 0 | 0 | 0 | n.a. | n.a. |
|  | 5 |  | 0 | 0 | 0 | n.a. | n.a. |
|  | 6 |  | 0 | 0 | 0 | n.a. | n.a. |
|  | 7 |  | 0 | 0 | 0 | 0 | n.a. |
|  | 8 |  | 0 | 0 | 0 | 0 | n.a. |
|  | 9 |  | 0 | 0 | 0 | 0 | n.a. |
|  | 10 |  | 0 | 0 | 0 | 0 | n.a. |
|  | 11 |  | 0 | 0 | 0 | 0 | n.a. |
|  | 12 |  | 0 | 0 | 0 | 0 | n.a. |
|  | 13 |  | 0 | 0 | 0 | 0 | 0 |
|  | 14 |  | 0 | 0 | 0 | 0 | 0 |
|  | 15 |  | 0 | 0 | 0 | 0 | 0 |
|  | 16 |  | 0 | 0 | 0 | 0 | 0 |
|  | 17 |  | 0 | 0 | 0 | 0 | 0 |
|  | 18 |  | 0 | 0 | 0 | 0 | 0 |
|  | 19 | Vehicle | 0 | 0 | 0 | n.a. | n.a. |
|  | 20 |  | 0 | 0 | 0 | n.a. | n.a. |
|  | 21 |  | 0 | 0 | 0 | n.a. | n.a. |
|  | 22 |  | 0 | 0 | 0 | n.a. | n.a. |
|  | 23 |  | 0 | 0 | 0 | n.a. | n.a. |
|  | 24 |  | 0 | 0 | 0 | n.a. | n.a. |
|  | 25 |  | 0 | 0 | 0 | 0 | n.a. |
|  | 26 |  | 0 | 0 | 0 | 0 | n.a. |
|  | 27 |  | 0 | 0 | 0 | 0 | n.a. |
|  | 28 |  | 0 | 0 | 0 | 0 | n.a. |
|  | 29 |  | 0 | 0 | 0 | 0 | n.a. |
|  | 30 |  | 0 | 0 | 0 | 0 | n.a. |
|  | 31 |  | 0 | 0 | 0 | 0 | 0 |
|  | 32 |  | 0 | 0 | 0 | 0 | 0 |
|  | 33 |  | 0 | 0 | 0 | 0 | 0 |
|  | 34 |  | 0 | 0 | 0 | 0 | 0 |
|  | 35 |  | 0 | 0 | 0 | 0 | 0 |
|  | 36 |  | 0 | 0 | 0 | 0 | 0 |

**Supplementary Table S7B. Safety analysis, clinical score data table of Hu-mice.** Clinical safety scores were established by characterization of five different clnical signs (coat, movement, activity, paleness, body weight), according to the following specification: Coat: score 0 – normal; score 1– lack of grooming, partial alopecia; score 2 – massive alopecia, wounds, bleedings, inflammation. Movement: score 0 – normal; score 2 – slow movement, paralysis of one animal; score 3 – difficulties to eat and drink, paralysis to more than one animal. Activity: score 0 – normal; score 1 – agitated, over-reactive, hypo-reactive; score 3 – prostrated. Paleness: score 0 – normal; score 1 – slight (no ear vessels visible); score 2 – severe (ears plus feet affected). Body weight: score 0 – normal; score 2 – segmentation of the vertebral column evident, pelvic bones palpable; score 3 – sceletal strucure prominent. Maximum cumulative clinical score allowed: 6. n.a.: not applicable.

| **Days after 1^st^ vaccination:** | | | **-7** | **-1** | **7** | **13** | **20** | **27** |
| --- | --- | --- | --- | --- | --- | --- | --- | --- |
| **Mouse strain** | **Mouse ID** | **treatment** | **Cumulative clinical score** | | | | | |
| Hu-mouse (Hu-NCG) | 37 | SARS-CoV-2 | 0 | 0 | 0 | 0 | n.a. | n.a. |
|  | 38 |  | 0 | 0 | 0 | 0 | n.a. | n.a. |
|  | 39 |  | 0 | 0 | 0 | 0 | n.a. | n.a. |
|  | 40 |  | 0 | 0 | 0 | 0 | n.a. | n.a. |
|  | 41 |  | 0 | 0 | 0 | 0 | n.a. | n.a. |
|  | 42 |  | 0 | 0 | 0 | 0 | n.a. | n.a. |
|  | 43 |  | 0 | 0 | 0 | 0 | 0 | n.a. |
|  | 44 |  | 0 | 0 | 0 | 0 | 0 | n.a. |
|  | 45 |  | 0 | 0 | 0 | 0 | 0 | n.a. |
|  | 46 |  | 0 | 0 | 0 | 0 | 0 | n.a. |
|  | 47 |  | 0 | 0 | 0 | 0 | 0 | n.a. |
|  | 48 |  | 0 | 0 | 0 | 0 | 0 | n.a. |
|  | 49 |  | 0 | 0 | 0 | 0 | 0 | 0 |
|  | 50 |  | 0 | 0 | 0 | 0 | 0 | 0 |
|  | 51 |  | 0 | 0 | 0 | 0 | 0 | 0 |
|  | 52 |  | 0 | 0 | 0 | 0 | 0 | 0 |
|  | 53 |  | 0 | 0 | 0 | 0 | 0 | 0 |
|  | 54 |  | 0 | 0 | 0 | 0 | 0 | 0 |
|  | 55 | Vehicle | 0 | 0 | 0 | 0 | n.a. | n.a. |
|  | 56 |  | 0 | 0 | 0 | 0 | n.a. | n.a. |
|  | 57 |  | 0 | 0 | 0 | 0 | n.a. | n.a. |
|  | 58 |  | 0 | 0 | 0 | 0 | n.a. | n.a. |
|  | 59 |  | 0 | 0 | 0 | 0 | n.a. | n.a. |
|  | 60 |  | 0 | 0 | 0 | 0 | n.a. | n.a. |
|  | 61 |  | 0 | 0 | 0 | 0 | 0 | n.a. |
|  | 62 |  | 0 | 0 | 0 | 0 | 0 | n.a. |
|  | 63 |  | 0 | 0 | 0 | 0 | 0 | n.a. |
|  | 64 |  | 0 | 0 | 0 | 0 | 0 | n.a. |
|  | 65 |  | 0 | 0 | 0 | 0 | 0 | n.a. |
|  | 66 |  | 0 | 0 | 0 | 0 | 0 | n.a. |
|  | 67 |  | 0 | 0 | 0 | 0 | 0 | 0 |
|  | 68 |  | 0 | 0 | 0 | 0 | 0 | 0 |
|  | 69 |  | 0 | 0 | 0 | 0 | 0 | 0 |
|  | 70 |  | 0 | 0 | 0 | 0 | 0 | 0 |
|  | 71 |  | 0 | 0 | 0 | 0 | 0 | 0 |
|  | 72 |  | 0 | 0 | 0 | 0 | 0 | 0 |

Supplementary Table S7C. Safety analysis, necropsy data table. Necropsy has been performed by macroscopic observation of spleen, liver, kidneys, stomach and intestine.

| **Mouse strain** | **Experimental day** | **Treatment** | **Necropsy result** |
| --- | --- | --- | --- |
| BALB/c | D14 | PolyPEPI-SCoV-2 | No abnormal observation in 6 of 6 |
|  |  | Vehicle | No abnormal observation in 6 of 6 |
|  | D21 | PolyPEPI-SCoV-2 | No abnormal observation in 6 of 6 |
|  |  | Vehicle | No abnormal observation in 6 of 6 |
|  | D28 | PolyPEPI-SCoV-2 | No abnormal observation in 6 of 6 |
|  |  | Vehicle | No abnormal observation in 6 of 6 |
| Hu-mouse  (Hu-NCG) | D14 | PolyPEPI-SCoV-2 | No abnormal observation in 6 of 6 |
|  |  | Vehicle | No abnormal observation in 6 of 6 |
|  | D21 | PolyPEPI-SCoV-2 | No abnormal observation in 6 of 6 |
|  |  | Vehicle | No abnormal observation in 6 of 6 |
|  | D28 | PolyPEPI-SCoV-2 | No abnormal observation in 6 of 6 |
|  |  | Vehicle | No abnormal observation in 6 of 6 |

# **REFERENCES**

Tarke, A., J. Sidney, C. K. Kidd, J. M. Dan, S. I. Ramirez, E. D. Yu, J. Mateus, R. da Silva Antunes, E. Moore, P. Rubiro, N. Methot, E. Phillips, S. Mallal, A. Frazier, S. A. Rawlings, J. A. Greenbaum, B. Peters, D. M. Smith, S. Crotty, D. Weiskopf, A. Grifoni, and A. Sette. 2021. 'Comprehensive analysis of T cell immunodominance and immunoprevalence of SARS-CoV-2 epitopes in COVID-19 cases', *Cell Rep Med*, 2: 100204.
